# Marsh migration and beyond: A scalable framework to assess tidal wetland resilience and support strategic management

**DOI:** 10.1371/journal.pone.0293177

**Published:** 2023-11-06

**Authors:** Rachel A. Stevens, Suzanne Shull, Jamie Carter, Emily Bishop, Nate Herold, Cory A. Riley, Kerstin Wasson

**Affiliations:** 1 Great Bay National Estuarine Research Reserve, New Hampshire Fish and Game Department, Greenland, New Hampshire, United States of America; 2 Padilla Bay National Estuarine Research Reserve, Mount Vernon, Washington, United States of America; 3 Office for Coastal Management, National Oceanic and Atmospheric Administration, Portland, Maine, United States of America; 4 Westward Ecology, Port Townsend, Washington, United States of America; 5 Office for Coastal Management, National Oceanic and Atmospheric Administration, Charleston, South Carolina, United States of America; 6 Elkhorn Slough National Estuarine Research Reserve, Watsonville, California, United States of America; 7 Department of Ecology and Evolutionary Biology, University of California, Santa Cruz, California, United States of America; University of South Florida, UNITED STATES

## Abstract

Tidal wetlands are critical but highly threatened ecosystems that provide vital services. Efficient stewardship of tidal wetlands requires robust comparative assessments of different marshes to understand their resilience to stressors, particularly in the face of relative sea level rise. Existing assessment frameworks aim to address tidal marsh resilience, but many are either too localized or too general, and few directly translate resilience evaluations to recommendations for management strategies. In response to the deficiencies in existing frameworks, we identified a set of metrics that influence overall marsh resilience that can be assessed at any spatial scale. We then developed a new comprehensive assessment framework to rank relative marsh resilience using these metrics, which are nested within three categories. We represent resilience as the sum of results across the three metric categories: current condition, adaptive capacity, and vulnerability. Users of this framework can add scores from each category to generate a total resilience score to compare across marshes or take the score from each category and refer to recommended management actions we developed based on expert elicitation for each combination of category results. We then applied the framework across the contiguous United States using publicly available data, and summarized results at multiple spatial scales, from regions to coastal states to National Estuarine Research Reserves to finer scale marsh units, to demonstrate the framework’s value across these scales. Our national analysis allowed for comparison of tidal marsh resilience across geographies, which is valuable for determining where to prioritize management actions for desired future marsh conditions. In combination, the assessment framework and recommended management actions function as a broadly applicable decision-support tool that will enable resource managers to evaluate tidal marshes and select appropriate strategies for conservation, restoration, and other stewardship goals.

## Introduction

Tidal marshes are critical components of coastal ecosystems. Wetlands protect the physical characteristics of coasts by buffering energy from storms and waves [1] while also providing habitat for nekton that support a broad suite of fish and wildlife, including wetland birds and economically significant fisheries species [2, 3]. Increasingly, these essential coastal features are under threat. Tidal marshes are facing anthropogenic stressors such as coastal squeeze, which is the reduction of habitat when sea level rise causes the shoreward boundary of a marsh to move inland while anthropogenic structures prevent equivalent migration of the landward edge, as human populations expand along coasts [4–6]. They also face impacts from climate change, including higher storm intensity and storm surge that cause increased flooding which may erode marsh borders. While increased sediment deposition during storms can offset erosion in some cases, in many places sedimentation rates may not keep up with the pace of relative sea level rise (RSLR) [7, 8]. Additionally, accelerated RSLR has the potential to reduce available habitat [9], change the composition of associated plant and animal communities [10, 11], and reduce the ability of tidal marshes to act as carbon sinks [12].

Strategic planning for the future of tidal marshes is critical in the face of RSLR. Wetlands have been managed for human activities for centuries, most commonly diked and drained to support farming or impounded for waterfowl hunting [13, 14]. Contemporary marsh conservation and restoration strategies more often focus on goals such as maintaining habitat values for the benefit of ecologically and economically significant species [15], protecting coastal infrastructure, mitigating storm damage [16], and sequestering greenhouse gas emissions by fostering conditions that enable wetlands to act as a carbon sink [17]. Many options exist to achieve these goals, and the strategy for different areas depends on the ultimate desired outcome. Managers may choose to prioritize protection of existing marshes through conservation efforts [13], or seek to improve the quality of existing wetlands through restoration or enhancement [18–20], or create new marshes where altered shorelines enable space for additional marsh habitats [21].

In order to conduct effective tidal marsh strategic planning in the face of accelerating RSLR, resource managers need a standardized approach to understand how particular marshes will respond to rapidly rising sea levels [22]. Not all marshes will respond equally to increasing RSLR; the current condition of tidal wetlands determines their vulnerability to outcomes such as excessive submergence when rising sea levels overtake a marsh’s accretion rate [23, 24]. Moreover, marshes vary in the challenges they face, in addition to or associated with RSLR, across local, regional, and national scales [25, 26]. For example, additional challenges may include nutrient loading, invasive species, or physical barriers like dams and dikes.

In addition to considering marsh vulnerability, management approaches to these challenges will vary depending on management goals. Some coastal managers may prioritize protection of particular plant or animal species [27, 28]. Others may determine that a particular tidal wetland is too degraded or faces too many challenges to be worth saving [29]. The cost to implement these strategies can differ considerably, so managers responsible for deciding where to allocate funds need science-based prioritization via a standardized assessment tool to evaluate and compare the resilience of marsh systems. This tool should be designed to provide management recommendations based on available data, and the outcomes of the marsh assessment should be linked to management actions.

A variety of resources are available to inform tidal marsh management decisions, yet no previously existing tool addresses all assessment needs. For example, the marsh resilience to sea level rise (MARS) multi-metric index focuses on future response by marshes to a single stressor, sea level rise, and does not evaluate either current condition or adaptation potential [30]. Similarly, the Unvegetated to Vegetated Ratio (UVVR) is a vulnerability metric developed as an indicator of sediment budget and vertical growth potential to predict future marsh condition, but does not account for availability or extent of migration pathways [31]. Several sophisticated marsh modeling techniques have been developed (e.g. MEM, WARMER, SLAMM) that are not always accessible for managers to employ on a local scale due to complexity or absence of required data inputs [32–35]. Conversely, many other studies are focused on a local or regional scale with metrics that are not applicable across geographic areas [36–38]. Many evaluation tools focus on marsh assessment and fail to provide recommended approaches to managers [15, 37, 39–41].

Our goal was to create an assessment framework that can be applied to tidal marshes at any spatial scale, from local to national or international, thereby enabling an apples-to-apples approach to comparing relative resilience of different marshes to RSLR in order to inform management decisions. We created an assessment framework wherein total marsh resilience is represented as the sum of scores across three metric categories: current condition, vulnerability, and adaptive capacity, and scores for any marsh within a study area represent resilience relative to other marshes included in the same analysis. We then linked marsh resilience scores directly to suggested actions to enable resource managers to target restoration and conservation projects strategically in key areas where project success and ecological impact will be maximized. Our new approach can thus be applied as a spatial planning tool at small or large geographic scales.

After developing the new framework, we applied it to 1,984 individual marsh units across the entire coastal contiguous United States (US). We included each of the 25 National Estuarine Research Reserves (NERRs) within this area, which collect data consistently and serve as an ideal platform for geographic syntheses [30, 42–44]. In addition, we summarize results for 8 hydrologic units within a single tidal marsh complex on the US Gulf Coast, where states in the southern US border the Gulf of Mexico, to examine smaller-scale patterns and associated recommendations for management. Overall, this framework functions as a decision-support tool that links relative resilience results to a variety of management options and enables cost-effective strategic planning decisions.

## Methods

Our objective was to create a tool that can be used by resource managers and coastal decision-makers to determine the best management options for tidal marshes at any spatial scale. We start by describing how the framework was developed, and then detail its scoring components and how the resilience scores link to management. Finally, to demonstrate use of this tool, we applied it at multiple geographic scales with publicly available national datasets and report on results within the contiguous US from regions, states, NERRs, and the Gulf Islands National Seashore, which includes sections of the Mississippi and Florida coastlines.

### Development of the framework

We considered site resilience using the definition adopted by the Nature Conservancy in their North Atlantic Landscape Conservation Cooperative resilience assessment model: “capacity of a physical site to maintain species diversity and ecological function even as the composition and proportion of habitats change in response to climate change” [38]. Guided by this definition, we then generated a comprehensive set of resilience metrics through a literature review and an expert elicitation process via a stakeholder survey completed by 34 staff members of the NERR system. During this process, we chose to focus on three critical components of resilience: current condition, adaptive capacity, and vulnerability. Available options for strategic spatial planning vary between categories, so we then solicited expert opinions on the most useful resilience metrics separately under each category from NERR staff during a session at the 2018 National Estuarine Research Reserve Association annual meeting. Next, we simplified the outcomes of the meeting to apply to all geographies and reviewed results with key members of the NERRs. We ultimately selected metrics that can be assessed in the US and internationally using publicly available data for vegetated tidal habitats, excluding mangrove systems. The list of metrics we provide can be expanded by users of this framework in areas where additional data are available. We intend the set of metrics within the framework’s three resilience categories to be flexible to the needs and resources of users. The resilience metrics outlined below are to be measured at the finest scale of interest and then aggregated at the marsh unit scale, defined as the smallest scale at which data for all metrics are available.

### Rationale behind categories and metrics

#### Current condition

The current condition of a marsh represents its relative starting point for ecosystem function before taking into account the effects of future RSLR (Table 1). The metrics selected for this category are based on measures of a marsh’s area compared to the length of its edge, the proportion of vegetated to unvegetated edge, and the proportion of surrounding impervious, agricultural, and natural land cover. Marsh configuration affects erosion exposure and geomorphology because a greater proportion of edges to core area, especially unvegetated edges, increase exposure to erosion [31, 42, 45–47]. Land cover and use is measured at the marsh unit scale within a small buffer to account for upland drivers of potential environmental stressors like runoff and pollution. Impervious and agricultural cover contribute runoff and pollutants that degrade marshes [48–51], while increased natural cover mitigates those effects [52–54].

**Table 1:** Resilience categories and metrics. [Note to printing production team: if possible, please separate the three broad categories in the final published table, either using gray shading for the first and third as we have done here, or by putting thick boundaries around each of the three categories, or a double line between the second and third].

Category	Group	Metric	±	Explanation	Refs
Current Condition	Marsh Configuration	Area to edge ratio	+	more core and less edge represents less exposure to erosion through wave action	[46, 55]
		Unvegetated edge ratio	-	more unvegetated edge represents more risk to tidal currents and RSLR	[31, 47, 56]
	Land Cover/Use in watershed	% impervious	-	more impervious can negatively impact runoff/habitat	[48, 49]
		% natural	+	more natural areas can absorb runoff/protect habitat	[52–54]
		% agricultural	-	more agriculture can negatively impact runoff/habitat	[50, 51]
Vulnerability	Erosion	Soil erodibility	-	high erosion potential represents greater risk of loss of sediment from marsh	[45, 57]
	Tides	Tidal range	+	a larger tidal range represents more resilience to RSLR, since plant elevational distribution is broader	[30, 32, 37, 58, 59]
		% marsh below MHHW	-	more marsh below MHHW represents more risk of converting high marsh to low marsh with RSLR	[24, 60]
		% marsh below MTL	-	more marsh below MTL represents more area at risk of excessive inundation in the face of RSLR	[45, 56, 61]
Adaptive Capacity	Shoreline	Hardened shoreline	-	more hardened shoreline represents more impediments to migration and decreased sediment supply	[4, 62, 63]
		Shoreline complexity	+	the more complex the shoreline the more opportunity for diverse habitats to support greater biodiversity	[38, 64, 65]
	Migration Ability	Marsh migration space	+	more migration space in low-lying uplands adjacent to marsh represents a larger potential future area for marsh to move into	[9, 35, 36, 66]
		Wetland "connectedness"	+	marshes that are more connected to each other in the future are able to support species migrations	[67]

Metrics are hierarchically nested within groups and categories. The ± column indicates whether a certain metric has a positive or negative influence on tidal marsh resilience to relative sea level rise (RSLR). MHHW refers to Mean Higher High Water, and MTL refers to Mean Tide Level.

#### Vulnerability to accelerating RSLR

These metrics evaluate how current marsh area will respond to increased stress from rapid RSLR (Table 1). Higher vulnerability is associated with lower overall resilience, and these metrics are calculated based on erodibility and elevation relative to tides. Marshes with higher erosion potential are less likely to keep up with RSLR, unless sediment inputs exceed erosion rates [45, 57]. Soil erosion could also increase with more frequent inundation [68]. Tidal metrics indicate how rapidly marshes will be affected by rising water levels [32], as marshes are more vulnerable to sea level rise when more of their vegetation is lower in the tidal frame, especially when that frame is not large [37, 58, 59]. A higher percentage of marsh below the Mean Tide Line (MTL) means more marsh consists of mudflat and higher percentage below the elevation of the highest tides translates to more low marsh elevation and associated vegetation [24]. Areas at risk of conversion from mud flat to open water, or low marsh to mud flat, in the face of accelerating RSLR may face marsh loss due to excessive submergence [45] or a loss of area to support low marsh plants that are limited by inundation and salinity ranges [60, 61]. RSLR comes from a combination of changes in the eustatic sea level driven by global climate change and changes in local isostatic sea level rise resulting from land subsidence in some areas, for instance from diking or groundwater overdraft [69]. Whether absolute water levels have risen, or the land has dropped, the two tidal marsh metrics account for more marsh area below the high tide line in the future. Metrics related to the elevational distribution of vegetation across a marsh [30] have been identified as robust indicators of observed marsh persistence [56].

#### Adaptive capacity

Exposure to wave energy, erosion, and inundation stress does not directly determine future condition, as tidal marshes may be able to expand laterally in response to RSLR. Adaptive capacity metrics address this point, as marshes with barriers to migration have lower resilience to RSLR [35]. These metrics are associated with shoreline features to indicate how easily the borders of a marsh can expand geographically and the ability for marshes to continue supporting ecological function (Table 1). The more sinuous, or complex, a shoreline, the more protection it affords a marsh [65] and more habitat niches exist to support a greater diversity of plants and animals [38, 64]. Hardened shorelines—which include rocky shores and built structures—impede migration [4, 62, 63]. The final metric group within this category, migration ability, addresses differences in land form and elevation that may help or hinder marsh area expansion [9, 36, 66]. The more connected a landscape is, the more capacity it has to support species that re-distribute in response to changing environmental conditions [67].

### Resilience scoring and links to management

After identifying the fundamental metrics and categories that contribute to tidal marsh resilience, we created a scoring system to sum results across categories. To generate scores, users of this framework first gather data associated with each metric for each marsh unit within an explicit spatial scale. Marsh units should be set by framework users based on the resolution of available data. Users then calculate decile breaks based on the spread of data for marshes within the chosen sample, which can be applied as cut points to create 10 equal bins within the raw data for each metric. Users then assign a rank score to each marsh unit relative to other marshes in the sample for each metric based on the corresponding bin (1–10) that contains the marsh units’ data value for a given metric. Next, users multiply metrics with a negative impact (as illustrated in Table 1) by negative 1, creating final scores for each metric. These scores represent the status of each marsh unit relative to the other marsh units assessed for each metric.

Users can add the individual metric scores together to create category resilience scores. All metrics are given equal weight when added, except hardened shoreline is weighted 1.5 times, and migration space is weighted 2 times to reflect the high impact of these metrics on tidal marsh resilience. Users should then calculate decile breaks by category for the summed metric scores and create 10 equal bins for each category based on the breaks. Users can assign rank scores to each marsh unit based on the corresponding bin (1–10) that contains the summed value for each marsh unit in each category. Users should assign a negative value to the category resilience scores for vulnerability to represent the negative impact of vulnerability on overall resilience. These scores represent the status of each marsh unit relative to the other marsh units assessed in each category.

Total resilience scores convert category resilience scores into a single metric that enables comparisons between marshes. To calculate total scores, users sum the three final scores for current condition, vulnerability, and adaptive capacity. Next, users calculate decile breaks for the metric scores summed across the categories and create 10 equal bins based on the breaks. Users then assign a rank to each marsh unit based on the corresponding bin (1–10) containing the summed value. These scores represent the overall resilience to RSLR of each marsh unit relative to the other marsh units assessed in a given analysis.

**Fig 1:**
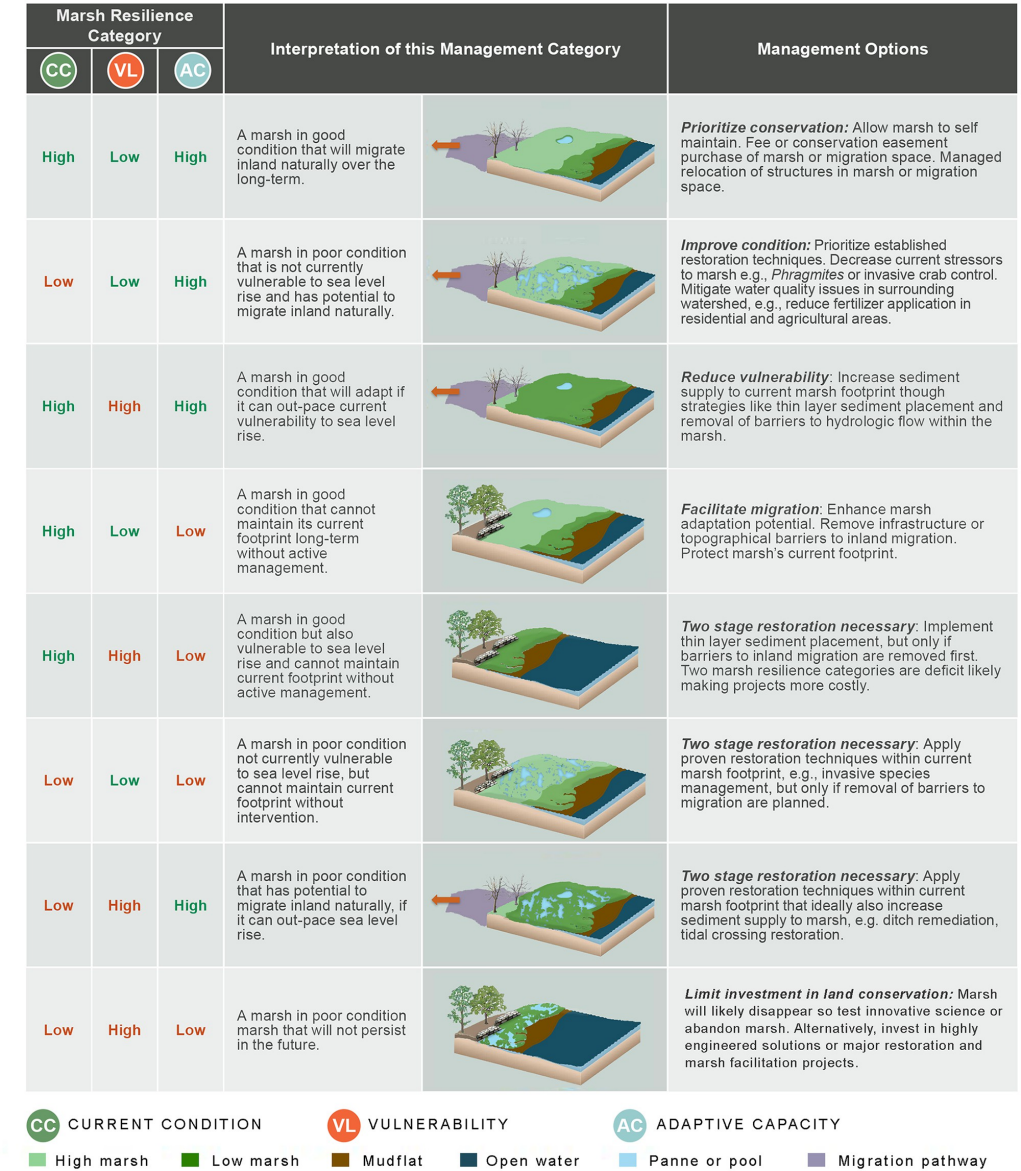
Best management options to maximize resilience to relative sea level rise for each marsh type. Green text indicates a positive condition, red reflects a negative one. From a restoration or adaptation practitioner’s perspective the more resilience categories that are shown in red for each marsh type, the more costly a project will likely be to implement.

To inform strategic planning, users can link category resilience scores to a series of management options. Users assign results for each marsh unit an ordinal category, either “low” or “high” based on their value. Category resilience scores less than 6 (greater than -6 for scores in the vulnerability category) are “low” while scores greater than or equal to 6 (less than or equal to -6 for scores in the vulnerability category) are “high”. All three categories are considered together to recommend best conservation or restoration actions, so users can generate a total management category using a concatenation of ordinal results within the three resilience categories. Finally, users are able to associate each total management category with a suite of management strategies that were developed during stakeholder meetings regarding restoration, conservation, and land use by policy professionals in New Hampshire (Fig 1). We simplified highly detailed locally relevant language from these meetings to be broadly applicable across a wide range of tidal marsh systems, then reviewed the final list of management options with key members of the NERR research, stewardship, and geographic information systems community.

### Application of the framework to US marshes

We calculated a total resilience score for each subwatershed hydrologic unit in the coastal contiguous US, and then summarized results across geographies to demonstrate the assessment framework’s value in comparing marshes at various spatial scales. We selected subwatersheds as our marsh unit for this analysis because that is the finest scale hydraulic unit in the Watershed Boundary Dataset, a data source from the US Geological Survey that provides polygon boundaries for summarizing surface water drainages within the entire US [70]. In the dataset, subwatershed hydraulic units are represented as 12 digit codes (HUC-12s), and are typically between 40 to 162 square kilometers [71]. We adopted these boundaries for our marsh units and used HUC-12 codes as Marsh Unit Codes (MUCs) for unique marsh unit identifiers. We provide additional details for metric calculation within each MUC in the Supporting Information (S1 File).

We compiled spatial data for current condition metrics from the National Oceanic and Atmospheric Administration (NOAA) Coastal Change Analysis Program (C-CAP) 2010 Regional Land Cover Data for the coastal United States [72]. We used the C-CAP 30 meter land cover data to identify estuarine wetland classes within MUCs and created a classification scheme to characterize core wetlands and edges. Using these data, we calculated a core-to-edge ratio [46], and a unvegetated to vegetated edge ratio inspired by the UVVR metric in development by [31] at the time of this analysis, for each 30 meter segment and then aggregated results for each MUC. We also used C-CAP to summarize development intensity for land cover metrics of percent impervious cover, natural cover, and agricultural cover with a 150 m buffer on each MUC. We selected this buffer size to match the land cover buffer used for tidal marsh monitoring in US National Wildlife Refuges [73].

We calculated soil erodibility using Esri’s USA SSURGO—Erodibility Factor imagery layer, which is 30 meter resolution, [74] and then aggregated and summarized by MUC. This layer draws upon on the US Department of Agriculture’s gNATSGO database [75] which is a compilation of data from walking surveys. We extracted tide datum data from NOAA’s VDatum tool [76] and interpolated data across gaps when any existed. Tidal range is the height difference between MHHW and Mean Lower Low Water (MLLW) measured in meters. The marsh elevations relative to tidal datums were determined using NOAA’s sea level rise lidar-based digital elevation model (DEM) [77] and tidal datums from NOAA’s VDatum grids. The lidar and tidal datum data were combined to create high resolution classified rasters depicting different tidal zonations. To measure percent of marsh below MHHW and below MTL, building on an earlier approach [30], we calculated the area below each level and divided values by the total marsh area in each marsh unit, then aggregated results by MUC. We used MHHW rather than MHW (Mean High Water), the tidal height used by an earlier assessment [30], to capture the maximum extent of tidal marsh transitional area. The underlying DEMs used are, with a few exceptions, gridded at a 3 meter horizontal resolution with a 0.328 feet (10 cm) or less root mean square error (RMSE) for vertical accuracy. NOAA’s VDatum model adds additional error (RMSE) to the base data that ranges from several centimeters to tens of centimeters depending on location. We further acknowledge that lidar data are often positively biased in marshes [78] but we did not attempt to remove this bias.

We used the NOAA Environmental Sensitivity Index (ESI) database [79] to calculate percent hardened shoreline and shoreline complexity for each marsh unit. We calculated migration space using NOAA’s sea level rise inundation data, and generated scores for each MUC based on projected inundation scenarios between 0.3 and 1.8 meters. Following an existing approach [36], within every MUC and for each foot of inundation above MHHW we divided the area of potential future marsh by the current area of marsh to generate a ratio of future to present "potential" marsh area. We then averaged the six scenarios for each MUC and generated a new percentile rank and score to create an average migration ratio metric. We computed wetland connectedness using MUC level data analyzed at a projected 1.2 meter sea level rise scenario, then used a region grouping process to group all connected marsh units under current and future scenarios. Within each MUC group, the number of unique future marsh units were subtracted from the number of unique current MUCs, and divided by the number of unique current MUCs. We used NOAA ESI shoreline vector data to characterize sinuosity within MUCs via a python script publicly available through Esri [80] using Esri ArcGIS software.

We followed the scoring framework outlined above to generate resilience scores by category and then calculated total scores to represent overall resilience for each marsh. We then summarized total resilience scores by averaging results across various scales (region/state/NERR) to demonstrate insights gained from large to medium scale application of this tool. Finally, we outline two case studies. In the first, we examined results from our national analysis on a marsh complex scale to represent the use of this framework within a single management area. We report metric and resilience scores for the 8 marsh units that make up the Gulf Islands National Seashore, a region on the Gulf Coast of the US that is preserved by the federal government for public recreation, in this example. This National Seashore includes islands in Florida and Mississippi, on either side of Mobile Bay, Alabama. In the second case study, to further demonstrate the how this framework can be downscaled for local decision making, we summarized its application by a conservation group in New Hampshire, with details provided in S3 File.

## Results

Here we provide illustrative results from implementation of the tidal marsh assessment framework within the contiguous US. Full results for each MUC are available in a geodatabase on NOAA’s Digital Coast website (S1 File) and summaries for each spatial scale detailed below are included in S2 File. On a national scale, 22% of the 1984 total MUCs meet the criteria for high priority for conservation as they are of good current condition, low vulnerability to RSLR, and have high adaptation so are likely to self-sustain into the future (Table 2). Of the marsh units we assessed, 17.5% are likely to not persist as they are of low current condition, are highly vulnerable to RSLR, and have low adaptation potential (Table 2).

**Table 2:** Summary of marsh resilience score results for tidal marsh units in coastal regions of the contiguous US. [Note to print production team: if possible, please using shading for alternate rows, as we have done here. Otherwise please ensure there is sufficient spacing between rows so that they can easily be distinguished. Also, please be sure not to replace the hyphens in the first column, both in the top row “Current Condition–Vulnerability–Adaptive Capacity” or in the subsequent ones “High–Low–High” etc. It is important to be clear that there are three separate scores, described in the top row and assessed in the subsequent ones].

Marsh Resilience Score	Region					Total
Current Condition- Vulnerability- Adaptive Capacity	Gulf of Mexico	Mid-Atlantic	Northeast	Southeast	West Coast	
High-Low-High	129	70	24	149	65	437
High-High-High	79	56	29	66	23	253
High-Low-Low	72	25	19	29	49	194
Low-Low-High	30	54	13	12	49	158
High-High-Low	86	37	14	9	13	159
Low-Low-Low	48	94	20	31	89	282
Low-High-High	22	63	26	23	19	153
Low-High-Low	71	154	77	26	20	348
Total	537	553	222	345	327	1984

Values represent the total number of Marsh Unit Codes (MUCs) with resilience scores corresponding to each marsh resilience category result. Marsh resilience scores are a concatenation of results for each resilience category: current condition, vulnerability, and adaptive capacity. Each unique combination of the three marsh resilience categories has a corresponding set of management recommendations, presented in Fig 1.

### Regional results

**Fig 2:**
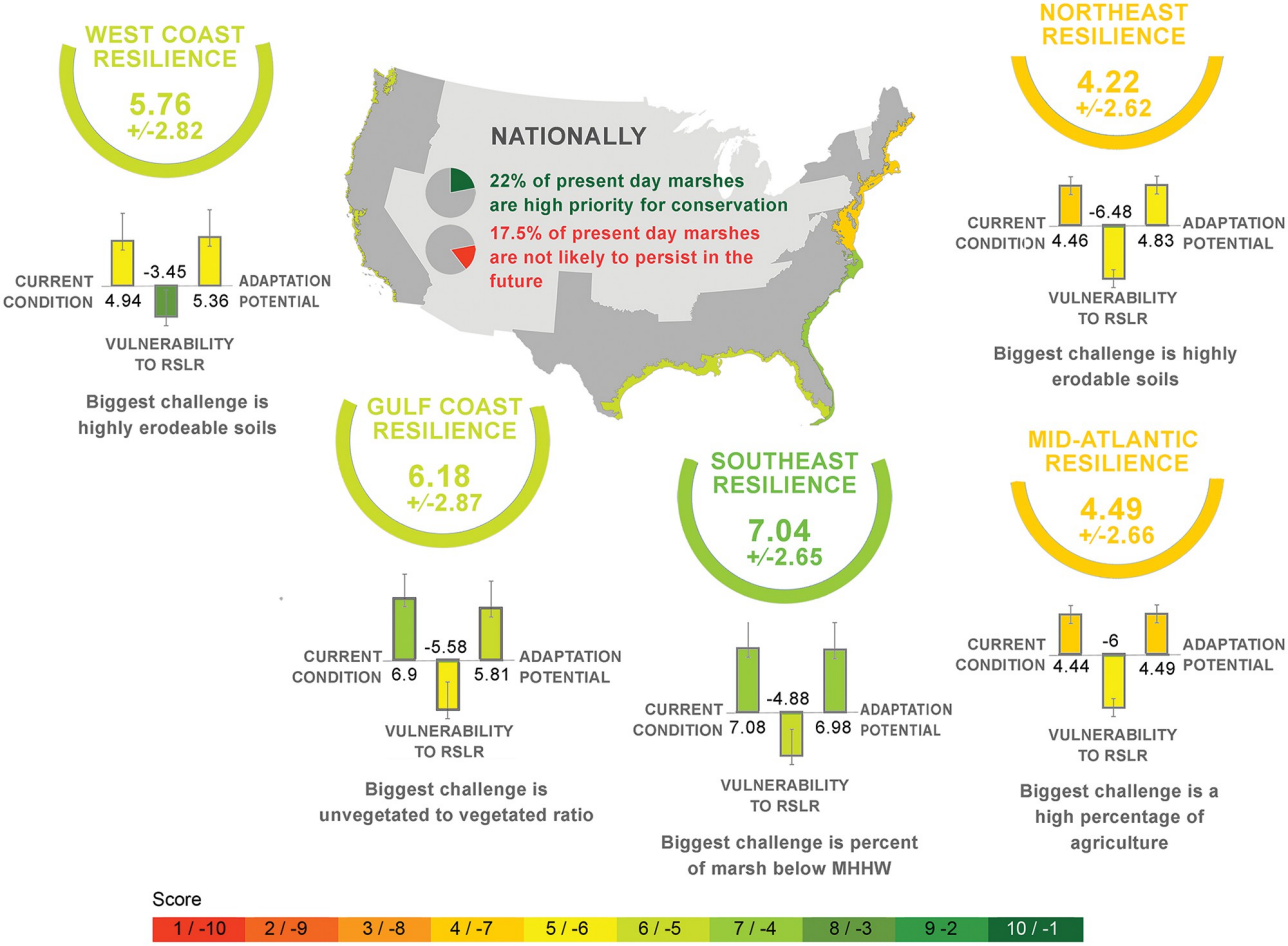
Regional resilience of marshes as assessed by metrics of current condition, vulnerability to relative sea level rise and marsh adaptation potential. The two categories that have a positive influence on tidal marsh resilience to relative sea level rise—current condition and adaptive capacity—range on a positive scale from 1 (low) to 10 (high). Collectively, metrics in the vulnerability category have a negative influence on resilience, so this category is scored on a negative scale from -1 (low vulnerability) to -10 (high vulnerability). MHHW stands for Mean Higher High Water. Country outline: United States Census Bureau.

Results of our analysis show that the Southeastern region of the US has the highest average total resilience score (7.04 ± 2.65 SD, Fig 2, S2 Table C in S2 File), while the Northeast has the lowest average total resilience score (4.22 ± 2.62). This is driven by the Southeast having the highest average current condition score (7.08 ±2.45), and average adaptive capacity score (6.98 ± 2.43). Conversely, the Northeast has the highest vulnerability, reflected as the most negative average vulnerability score (-6.48 ± 2.59) and the lowest average adaptive capacity score (4.83 ±2.65). While the Gulf of Mexico also has a high average current condition score (6.9 ± 2.67), the West Coast, Mid-Atlantic, and Northeast have relatively equal low average current condition scores (4.44–4.94). The Mid-Atlantic also ranked high in average vulnerability (-6.00 ±2.71) while the West Coast is ranked the least vulnerable on average (-3.45 ±2.95).

The biggest challenge, or worst score within any category, varied by region. The Gulf of Mexico is limited by a high unvegetated edge to edge marsh ratio, while the Mid-Atlantic has a high percentage of agricultural area (S2 Table C in S2 File). The Northeast and West Coast are both limited by high erodibility, while the Southeast is limited by percent marsh below MHHW. Conversely, along the West Coast and Northeast large tidal ranges were a positive influence. In the Gulf of Mexico, large unfragmented marsh blocks (a high core to edge ratio) strengthened resilience. Connectedness of tidal wetlands had the most beneficial effect of any factor in both the Southeast and Mid-Atlantic (S2 Table C in S2 File).

### Results by state

Consistent with the regional analysis, the least resilient states were in the Northeast. New York, Connecticut, and Rhode Island scored lowest in average total resilience (1.95 ± 1.70, 2.21 ± 2.25, 2.78 ± 2.2, Table 2, S2 Table D in S2 File). Rhode Island also scored worst in average marsh current condition (2.37 ± 1.80), as did New York in the adaptive capacity category (1.93 ± 1.79). Mississippi scored most vulnerable on average (-8.07 ± 2.63). Marshes in the Southeastern states of Georgia, South Carolina, and Florida had the highest average total resilience scores (8.54 ± 1.45, 7.64 ± 1.92, 7.09 ± 2.85). Georgia also ranked highest in average current condition, as did South Carolina in adaptive capacity (7.41 ± 1.98), while California scored least vulnerable on average of any state (-1.89 ±1.48).

The soil erodibility metric, in the vulnerability category, was the most negative factor for several states (CT, ME, MI, NH, OR, TX, WA), followed by percent impervious surface (CA, FL, MA, NJ, NY, RI, see results in S2 File). The most common positive factor was mean marsh tidal range (CA, CT, GA, MA, ME, NH, NJ, NY, OR, PA, RI, WA) followed by tidal wetland connectedness (AL, DE, FL, MD, SC, see full results in S2 Table D in S2 File).

### Results by NERR

**Fig 3:**
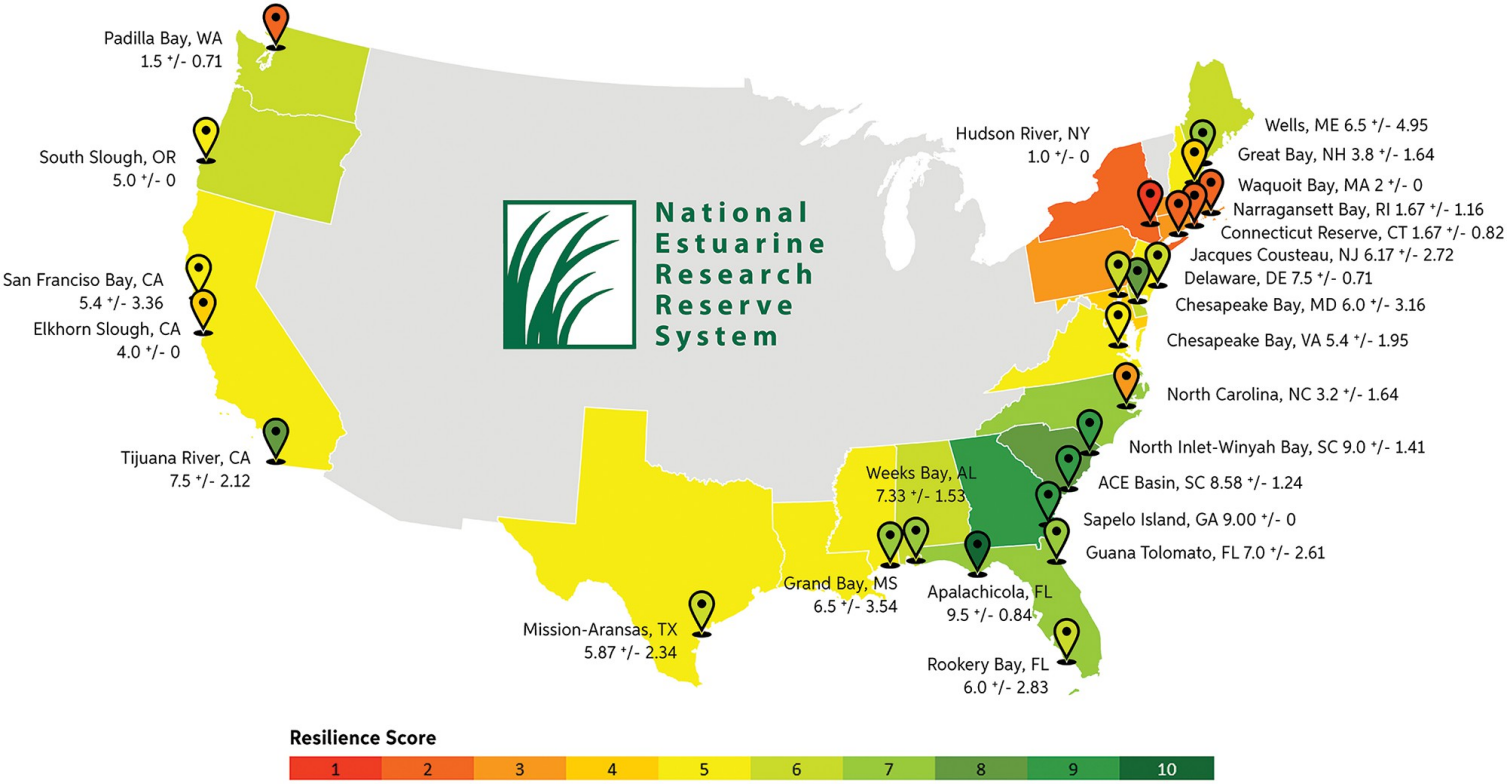
Average marsh resilience score for each coastal state and each National Estuarine Research Reserve within the contiguous US. Reserves with a standard deviation of zero have their boundaries contained all within one HUC-12 boundary. Country outline: United States Census Bureau.

We evaluated resilience for all 25 NERRs in the coastal contiguous US (Fig 3). The NERR with the lowest current condition is Padilla Bay in Washington State and in contrast to the regional trend for the West Coast, this reserve is also the most vulnerable. Hudson River, New York ranks lowest in adaptive capacity and overall resilience (S2 File and Fig 3), which aligns with the Northeast regional trend. The highest current condition NERRs were in the Southeast region (North-Inlet Winyah Bay, South Carolina and Sapelo Island, Georgia) as were the least vulnerable (Rookery Bay, Florida) and best adaptive capacity and overall resilience (Apalachicola Bay, Florida, S2 Table E in S2 File). Tijuana River, California also ranked highly in adaptive capacity (S2 Table E in S2 File).

The most common metrics reducing resilience were related to land cover, with the top two being percent impervious surface (7 reserves) and percent agriculture (6 reserves). Percent of marsh below MHHW was also a common negative factor (6 reserves). The most common positive factors were marsh core-to-edge ratio (12 reserves) and mean tidal range (8 reserves, S2 Table E in S2 File).

### Case study: Gulf Islands National Seashore

**Fig 4:**
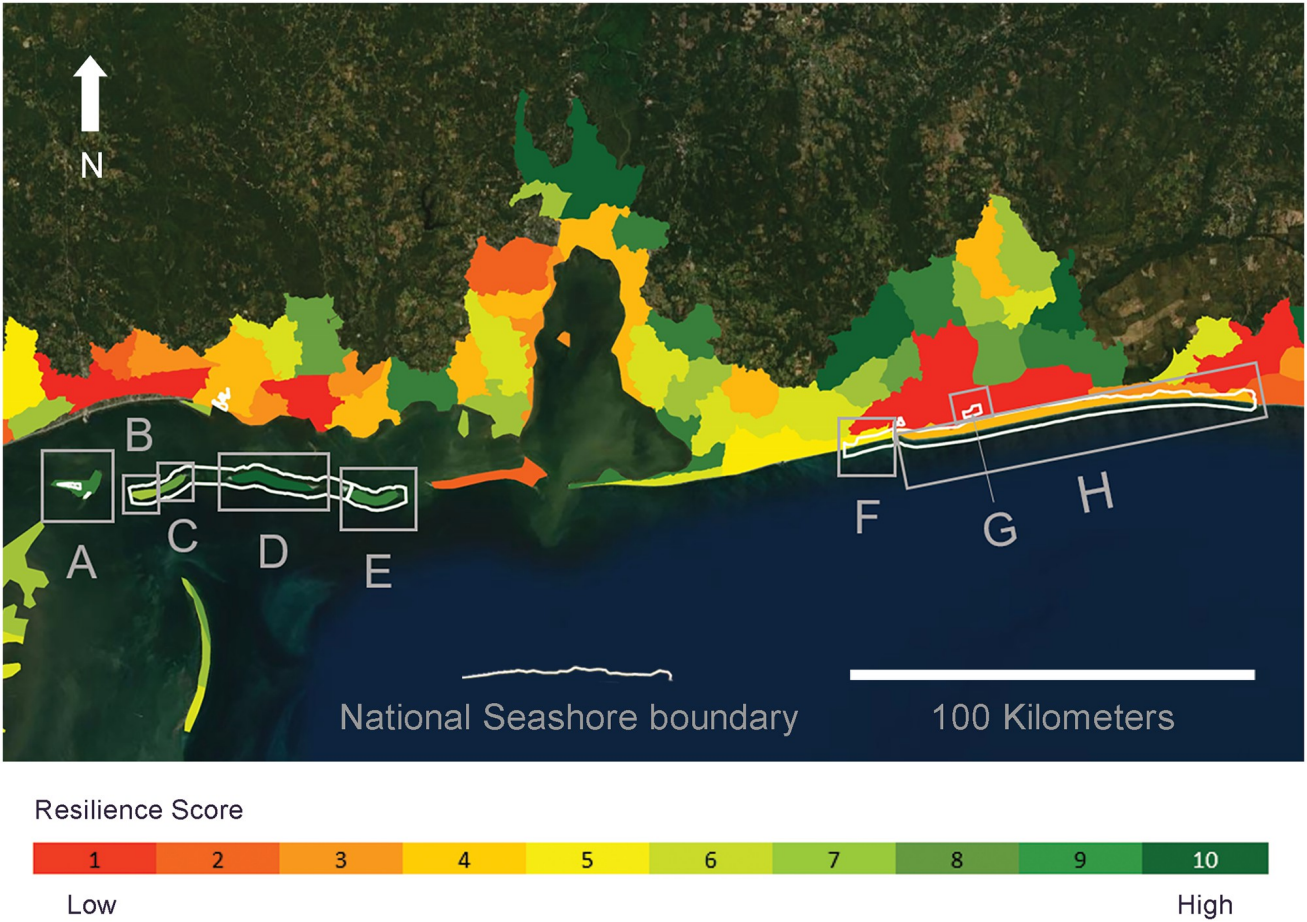
Resilience scores for marsh units of the Gulf Islands National Seashore in Mississippi and Florida. Colored polygons represent MUCs assessed in our national application of the framework. Islands labeled with letters are individual subwatershed boundaries within the Gulf Islands National Seashore. Country outline: United States Census Bureau. World Imagery source: Esri, DigitalGlobe, GeoEye, i-cubed, USDA FSA, USGS, AEX, Getmapping, Aerogrid, IGN, IGP, swisstopo, and the GIS User Community.

**Fig 5:**
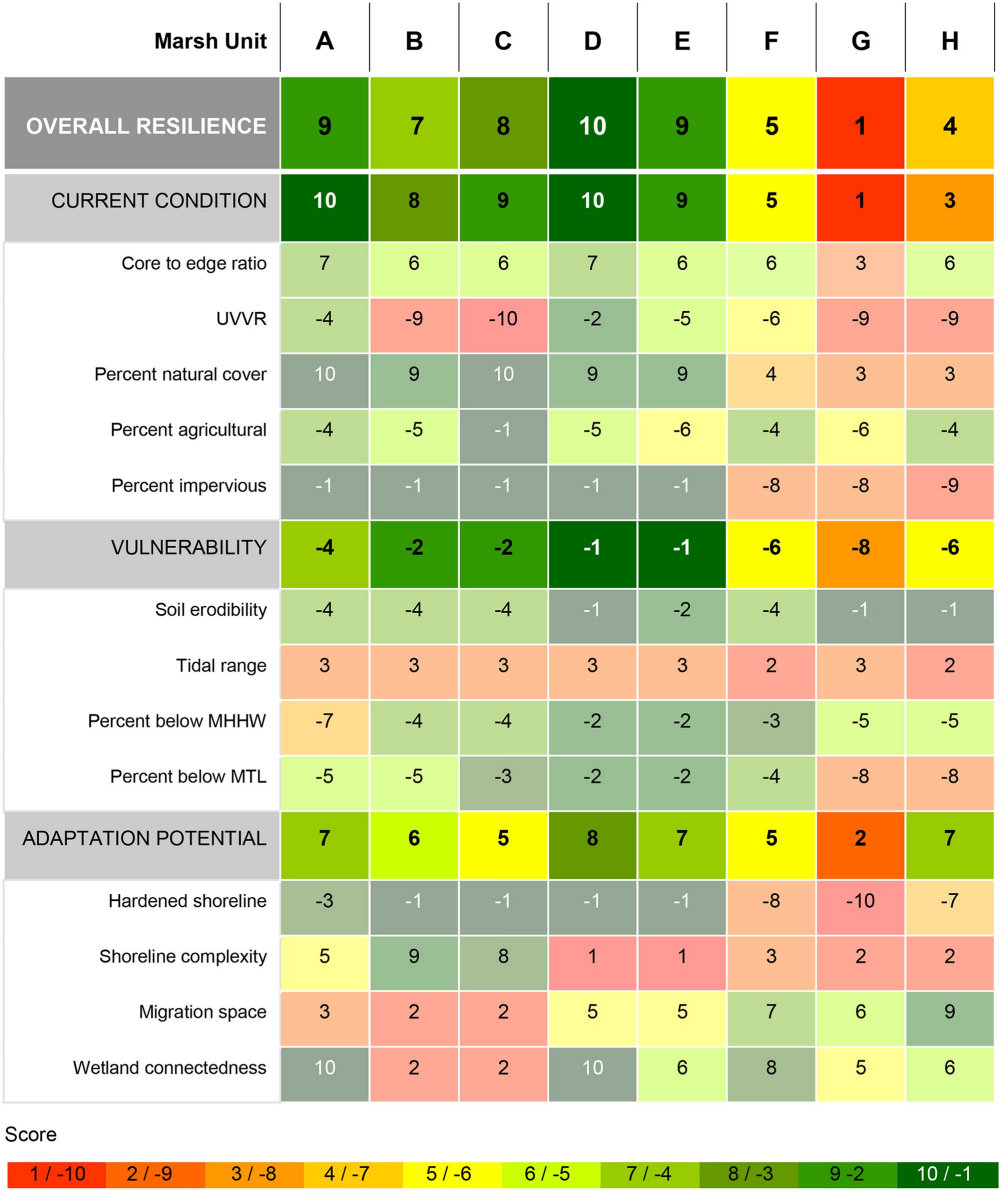
Resilience scores for each marsh unit, resilience category, and composite metric in the Gulf Islands National Seashore. Letters identify marsh units within the National Seashore, as displayed in Fig 4. The two categories that have a positive influence on tidal marsh resilience to relative sea level rise—current condition and adaptive capacity—range on a positive scale from 1 (low) to 10 (high). Collectively, metrics in the vulnerability category have a negative influence on resilience, so this category is scored on a negative scale from -1 (low vulnerability) to -10 (high vulnerability). This figure serves as an example of detailed results generated by applying our tidal marsh assessment framework.

The eight marsh units in the Gulf Islands National Seashore span almost 2,500 square kilometers of Mississippi and Florida coastline on either side of Mobile Bay, Alabama (Fig 4). We present detailed results from these MUCs as a demonstration of the applicability of our assessment framework. The average resilience score for the entire National Seashore is 6.62 (± 3.07), driven by moderate average scores in each of the three metric categories (6.88 ±3.44 current condition, -4.00 ± 2.20 vulnerability, 5.88 ±1.88 adaptive capacity). Individual metric scores vary significantly between the MUCs in Mississippi to the west and Florida to the east (Fig 5). The average total score for marsh units in the western portion of the National Seashore (marsh units A-E) is 8.60 (± 1.14) while the average total resilience score for marsh units in the eastern portion (marsh units F-H) is 3.33 (± 2.08). While these two sides of the National Seashore have relatively similar average adaptive capacity scores (6.60 ± 1.14 for Mississippi and 4.67 ± 2.52 for Florida), their average current condition scores differ considerably (9.20 ± 0.84 for Mississippi and 3.00 ± 2.00 for Florida).

## Discussion

We combined resilience categories, each composed of multiple resilience metrics, with management recommendations in a framework that can be used to evaluate and compare tidal wetlands worldwide. The framework can be used to examine overall marsh resilience, or a single or subset of metrics. Many of the resilience metrics can be mitigated by management strategies (e.g., percent impervious surface) while others cannot (e.g., tidal range). Understanding the contribution of individual metrics to overall resilience is valuable when developing strategic priorities aimed at addressing relevant mechanisms. Robust datasets from authoritative sources (e.g., C-CAP) facilitated the assessment of marshes across a broad geographic scale, the entire coastal contiguous US. As these datasets are updated, the marsh assessments can be updated as well.

There are several limitations to our study. We did not conduct validation on the framework as a whole but this would be a valuable project in the future, following an established approach [56]. Future efforts could also include consideration of mangroves, another critical coastal wetland system that is susceptible to RSLR. Our current analysis is also limited in that we do not include a metric to account for sediment supply, which is a factor in vertical migration potential [81]. However, if future users of this framework have sediment supply data available for their area of interest, that metric could be easily incorporated. Evaluating sediment supply in combination with tidal range metrics is likely most valuable within regions, because variability in geomorphology complicates generalizations across regions [56]. The results of our analysis at the national scale lack the precision afforded by methods based on field data collection [82] but are a valuable extension of previous efforts to characterize tidal marsh resilience across geographies that relied on interpolating results between far fewer data points [30, 83, 84].

### Trends in tidal marsh resilience categories

Our development of the three categories in this framework improves our ability to evaluate and compare relative resilience across marshes.

#### Current condition

We developed a straightforward method to characterize the current condition of any tidal marsh by selecting assessment metrics that are applicable across geographies. Previous studies have developed methods to evaluate marsh condition on a local or state-wide scale (see [14, 85–87] for examples), but methods and metrics that are highly relevant locally do not always translate to broad-scale comparisons between marshes. For example, percent cover of brackish border species, a vegetation metric used to indicate human disturbed marshes in the Northeast, would not be an indicator for degraded marsh in other regions with naturally lower salinities [83]. Current condition scores from the broadly applicable metrics in our framework agreed with a sampling method to assess the current condition of small marshes ultimately determining that Rhode Island tidal marshes are degraded [36]. This conclusion, based on data collected in the field, aligns with our findings, based on remote sensing, that RI had the lowest current condition of any state. Our assessment also found that the Northeast ranked low in current condition overall, which agrees with an analysis that found that marshes in this region were already experiencing declines in response to RSLR [88].

#### Vulnerability

We adopted a robust approach to represent marsh vulnerability that is easier to apply than complex models though our results in this category vary from modeled results to different degrees. Many methods have been used to evaluate vulnerability, particularly in the last decade when accelerated RSLR has become a growing concern. For example, the West Coast was considered highly vulnerable due to low accretion rates and limited migration space [84], while we found that the West Coast is the least vulnerable region relative to others within the contiguous US based on erodibility, tidal range, and area above mean tide levels. An earlier investigation found that the mid-Atlantic is highly vulnerable relative to the Mississippi River and Texas Gulf [89], which aligns with our results that the mid-Atlantic is more vulnerable than the Gulf of Mexico. Their conclusion resulted from differentials in sediment supply between regions. As previously mentioned, our current analysis did not account for sediment supply, however our vulnerability assessment also reached the same conclusion as another study that assessed sediment supply and predicted the Southeast to be less vulnerable to RSLR than the Northeast [60].

#### Adaptive capacity

Our framework accounts for previously overlooked factors that contribute to future marsh conditions. Until lately, adaptive capacity has received the least attention in assessing tidal marsh resilience though there have been several recent broad scale assessments [9, 66, 90–92]. Our approach is similar to others that utilize remote sensing techniques as a robust measure of migration space; for example, low resilience in the Mid-Atlantic was linked to limited available area for landward retreat in addition to limited vertical accretion potential [41]. We found that the Mid-Atlantic scores about average for migration ratio compared to other regions and that no one metric stands out as contributing to, or detracting from, resiliency in this region. We rank the West Coast overall above the Northeast and Mid-Atlantic in adaptive capacity driven in part by a lower percent armored shoreline and a higher average migration ratio. Our results for the Northwest align with an assessment that determined that while that region may be highly vulnerable, it is not the most vulnerable region within the contiguous US [41]. Another study characterized the Gulf Coast as highly vulnerable to RSLR, but identified significant areas available for marsh migration [66]. We found the Gulf Coast to be one of the more resilient regions, driven in part by higher scores in current condition metrics and variable, but about average, migration ratio scores.

### Linking assessment to strategic planning

We provide a framework for applying metrics that can be used as a screening tool to select the most appropriate management practices for a specific site. Coastal managers need an approach that integrates robust metrics [56] to focus on increasing resilience [13], which our study achieves at multiple spatial scales. The decision support table associated with our resilience scoring can be used with most data sources that characterize tidal marsh status within the three metric categories, so Fig 1 could be applied in areas with different data sources. This approach allows managers to identify the most cost-effective projects to achieve strategic planning goals. For example, marshes with low adaptive capacity scores could be good candidates for restoration projects that remove barriers to migration. If the same marsh has a high vulnerability to sea level rise, experimental techniques like thin layer placement (TLP) may also be appropriate. TLP raises the level of the marsh plateau, decreasing its vulnerability to sea level rise. A strategy like this may be suited to restoring Marsh G in our case study example, which has a moderate current condition score. Furthermore, a non-profit or government agency aiming to improve resiliency of tidal marshes in the Southeast and Gulf Coast regions may link scores from our analysis to the management recommendations in Table 1 to conclude that investments should be directed towards protections for existing marshes in Georgia, removal of barriers to migration in some areas in Louisiana, and creation of new higher marsh plains in Alabama.

Comparing resilience categories can be enhanced by looking more closely at individual metrics most relevant to the management option under consideration. For example, the portion of marsh below MHHW could be used to identify marshes with high-high-high composite resilience scores in that are within an elevation range optimal for TLP. A marsh that is almost all below MHHW, such as the average marsh unit within the South Slough NERR in Oregon, might need a large amount of additional sediment to keep up with sea level rise, making TLP less appropriate than thick-layer sediment addition. Another site that is almost all above MHHW, such as the Weeks Bay NERR in Alabama, may only need a small amount of sediment to reduce vulnerability and therefore TLP may be more feasible. The management recommendations we provide serve as a broadly applicable starting point for planning purposes, though individual sites will need further evaluation and consideration.

### Scalability of new framework

Our framework can be applied at the estuary or watershed level across tidal marshes on a national and international scale. While the scores generated through this framework should not be used as absolute ranks to compare resilience to RSLR across studies, they are a valuable tool for comparing marshes analyzed within a study. By applying the framework to tidal marshes throughout the nation, we found that the most commonly recommended management option linked to resilience scores in the contiguous US is to maintain current marsh extent through conservation actions (Figs 1 and 2). This is primarily driven by the Gulf of Mexico and Southeast, which were the regions with the highest average total resilience scores (Fig 2). These areas are characterized by higher core to edge ratio, higher percent natural watershed, and lower erodibility than other regions we assessed. The second most commonly recommended management category was to limit protection of existing vegetation because of low overall resilience, with The Mid-Atlantic and Northeast contributing the majority of total marsh units with this condition (Table 2). Marshes in these regions are candidates for investments in major restoration and marsh facilitation projects including removal of barriers to migration, and they may also offer opportunities to test experimental restoration methods.

Resilience metrics analyzed within our framework can be used to evaluate how well a specific marsh represents the broader system in which it is located, or to see if the spatial design of a research or monitoring program represents the range of conditions in the study area. For example, in our case study all but one of the marsh units in the Gulf Islands National Seashore score well for the percent of marsh below MHHW, so any marsh unit besides Marsh Unit A can be considered representative of the National Seashore’s overall marsh elevation (Fig 5). The value of individual metrics can also vary across scales, for example although tidal range varies between regions within the US, it is fairly consistent within local geographies. In contrast, land cover metrics such as UVVR and hardened shoreline can be locally heterogeneous. In cases where high resolution data are available, on a plot or transect scale, this framework can be applied to compare areas within a single tidal marsh. Additional metrics can also be added within any of the three resilience categories to expand assessments wherever data are available across the area of interest. For example, we used a downscaled application of this framework to support local conservation planning in the US state of New Hampshire, adding metrics to the current condition category in response to stakeholder needs (S3 File).

Multi-component regions or reserves can benefit from considering how resilience scores differ among sites and how that may inform experimental design, infrastructure placement, or priority areas to acquire for conservation. In our primary case study example, marsh units at the western end of the Gulf Islands National Seashore will require less investment than marsh units on the eastern side for increased resilience to RSLR (Fig 4). Interventions that are specific to an area of management concern may benefit more than one marsh unit, so identifying local trends could enhance efficiency of restoration investments. For example, adjacent marsh units on the eastern side of our Gulf Islands National Seashore case study may all be good candidates for actions that increase sediment supply to the marsh (Figs 4 and 5). While our application of this framework at the national level supports large-scale planning and can serve as a screening tool for smaller scales, local and regional planners would benefit from applying the framework within their specific area of interest, with the finest resolution data available for that scale, to maximize the precision of local management recommendations. In combination, individual metrics, total resilience scores, and linked management categories provide critical information for resource managers to proactively plan for tidal marsh response to RSLR at the local and landscape scale.

## Supporting information

S1 File Geospatial metadata for calculating tidal marsh resilience metrics. (PDF)

S2 File Summary results for each spatial scale of application to US marshes. (PDF)

S3 File Case study parcel scale planning in New Hampshire. (PDF)
