# Supplementary material for: Marsh migration and beyond: A scalable framework to assess tidal wetland resilience and support strategic management
Source: PLoS One. 2023 Nov 6;18(11):e0293177. doi: 10.1371/journal.pone.0293177 (PMC10627444; doi:10.1371/journal.pone.0293177)
Supplement: S1 File — (PDF) [file pone.0293177.s001.pdf]

S1. Geospatial metadata for calculating tidal marsh resilience metrics. All geospatial metadata for the application of this tidal marsh resilience framework in the coastal contiguous United States is available on the NOAA InPort NMFS Enterprise Data Management Program site at https://www.fisheries.noaa.gov/inport/item/62985. A copy of this webpage is also included below as a static archive. Results from the application of these methods, titled "Tidal Marsh Resilience to Sea Level Rise," are available for download as a geodatabase on the NOAA Office for Coastal Management Digital Coast website at https://coast.noaa.gov/digitalcoast/data/marshresilience.html and as an Excel file on Github at https://github.com/e-bish/Tidal_Marsh_Resilience. All of the code used to generate these results is available in the Github repository in both Python and in R languages. We also provide an Excel file that additionally replicates the calculations to generate the same results. A story map to visualize the results, titled "Salt Marsh Resilience to Sea Level Rise" can also be found on arcgis.com at (arcgis.com)https://experience.arcgis.com/experience/25037478b4634bb2bf421f443ac47541/page/page_0/?views=view_6%2Cview_19%2Cview_24.

OCM Metadata Library > Raster > C-CAP >

Salt Marsh Resilience, National, 2010

Data Set (DS) | Office for Coastal Management (OCM)

GUID: gov.noaa.nmfs.inport:62985 | Updated: August 9, 2022 | Published / External

COMPLETION RUBRIC
69%
24.2 / 35
View Report

View As | View in Hierarchy

Short Citation:
Office for Coastal Management, 2023: Salt Marsh Resilience, National, 2010, https://www.fisheries.noaa.gov/inport/item/62985.

Full Citation Examples

Item Identification

**Title:**	Salt Marsh Resilience, National, 2010
**Status:**	Completed
**Publication Date:**	2020-09-25
**Abstract:**	This polygon data set includes raw values and normalized scores for thirteen landscape scale metrics that characterize marsh resilience to sea level rise within watersheds along the coast of the conterminous United States. These metrics fall into bins related to current marsh condition, marsh vulnerability, and adaptation potential. The data are summarized at the watershed scale (HUC-12 units).
**Purpose:**	This data set was developed to support a protocol to assess tidal marsh resilience at the landscape scale by using GIS-based metrics of current marsh condition, vulnerability to sea level rise, and potential for adaptation. The protocol supports standardized comparisons of marsh conditions over large areas along the coasts and within the National Estuarine Research Reserve System (NERRS). Used in tandem with other NERRS-based marsh assessment tools, it can provide an integrated continuum of assessment to inform efforts to study, restore, or protect tidal marshes at the local, state, regional, and national scales.
**Other Citation Details:**	NOAA Office for Coastal Management, National Estuarine Research Reserve System, Great Bay NERR, Padilla Bay NERR, University of New Hampshire

Supplemental Information: Vulnerability scores were updated to fix an error caused by the erroneous inclusion of the hardened shoreline metric in the original scoring (3/23/2022).

Keywords

Theme Keywords

Thesaurus	Keyword
Global Change Master Directory (GCMD) Science Keywords	EARTH SCIENCE > LAND SURFACE > LAND USE/LAND COVER
Global Change Master Directory (GCMD) Science Keywords	EARTH SCIENCE > LAND SURFACE > LAND USE/LAND COVER > LAND USE/LAND COVER CLASSIFICATION
**UNCONTROLLED**	
Global Change Master Directory (GCMD) Instrument Keywords	Earth Remote Sensing Instruments > Passive Remote Sensing > Photon/Optical Detectors > Cameras > CAMERAS
Global Change Master Directory (GCMD) Platform Keywords	Aircraft > AIRCRAFT
ISO 19115 Topic Category	imageryBaseMapsEarthCover
None	Biota
None	Digital Coast
None	Lidar
None	National Estuarine Research Reserves
None	NERRS
None	NOAA
None	Remotely Sensed Imagery/Photos
None	Resilience
None	Salt Marsh

None	Sea Level Rise
None	Wetlands

Spatial Keywords

Thesaurus	Keyword
**UNCONTROLLED**	
Global Change Master Directory (GCMD) Location Keywords	Ocean > North America > United States of America
None	Coastal Zone

Physical Location

**Organization:**	Office for Coastal Management
**City:**	Charleston
**State/Province:**	SC

Data Set Information

**Data Set Scope Code:**	Data Set
**Maintenance Frequency:**	Unknown
**Data Presentation Form:**	File Geodatabase
**Distribution Liability:**	Users must assume responsibility to determine the usability of these data.

Support Roles

Data Steward

CC ID: 989526

**Date Effective From:**	2020-09-25
**Date Effective To:**	
**Contact (Organization):**	NOAA Office for Coastal Management (NOAA/OCM)
**Address:**	2234 South Hobson Ave<br>Charleston, SC 29405-2413
**Email Address:**	coastal.info@noaa.gov
**Phone:**	(843) 740-1202
**URL:**	https://coast.noaa.gov

Distributor

CC ID: 989528

**Date Effective From:**	2020-09-25
**Date Effective To:**	
**Contact (Organization):**	NOAA Office for Coastal Management (NOAA/OCM)
**Address:**	2234 South Hobson Ave<br>Charleston, SC 29405-2413
**Email Address:**	coastal.info@noaa.gov
**Phone:**	(843) 740-1202
**URL:**	https://coast.noaa.gov

Metadata Contact

CC ID: 989529

**Date Effective From:**	2020-09-25
**Date Effective**	

**To:**	
**Contact (Organization):**	NOAA Office for Coastal Management (NOAA/OCM)
**Address:**	2234 South Hobson Ave<br>Charleston, SC 29405-2413
**Email Address:**	coastal.info@noaa.gov
**Phone:**	(843) 740-1202
**URL:**	https://coast.noaa.gov

Point of Contact

CC ID: 989527

**Date Effective From:**	2020-09-25
**Date Effective To:**	
**Contact (Organization):**	NOAA Office for Coastal Management (NOAA/OCM)
**Address:**	2234 South Hobson Ave<br>Charleston, SC 29405-2413
**Email Address:**	coastal.info@noaa.gov
**Phone:**	(843) 740-1202
**URL:**	https://coast.noaa.gov

Extents

**Currentness Reference:**	Publication Date

Extent Group 1

**Extent Description:**	Data cover the coastal watersheds of the conterminous United States that contain estuarine wetland cover types.

Extent Group 1 / Geographic Area 1

CC ID: 989540

**W° Bound:**	-127.854
**E° Bound:**	-65.362
**N° Bound:**	51.534
**S° Bound:**	22.885

Extent Group 1 / Time Frame 1

CC ID: 989539

**Time Frame Type:**	Range
**Start:**	2009-01-20
**End:**	2011-11-11

Spatial Information

Spatial Representation

Representations Used

**Grid:**	No

Reference Systems

Reference System 1

CC ID: 989932

Coordinate Reference System

**CRS Type:**	Projected
**EPSG Code:**	EPSG:5070
**EPSG Name:**	NAD83 / Conus Albers

▾ See Full Coordinate Reference System Information

Access Information

**Security Class:**	Unclassified
**Data Access Constraints:**	None
**Data Use Constraints:**	Data set is not for use in litigation. While efforts have been made to ensure that these data are accurate and reliable within the state of the art, NOAA, cannot assume liability for any damages, or misrepresentations, caused by any inaccuracies in the data, or as a result of the data to be used on a particular system. NOAA makes no warranty, expressed or implied, nor does the fact of distribution constitute such a warranty.

URLs

URL 1

CC ID: 989531

**URL:**	https://coast.noaa.gov/htdata/raster1/landcover/bulkdownload/hires
**URL Type:**	Online Resource

URL 2

CC ID: 989532

**URL:**	https://coast.noaa.gov/digitalcoast/data/ccaphighres
**URL Type:**	Online Resource

Technical Environment

Description: Microsoft Windows XP Version 5.1 (Build 2600) Service Pack 3; ESRI ArcCatalog 9.2.2.1350

Data Quality

Conceptual Consistency:

Data are complete and cover all areas defined by the source data.

Standard mapping methods were used to develop the data, which are described in the Process Steps section. Outputs were inspected for errors and inconsistencies, which were addressed and remedied.

Marsh resilience quality assurance review.

Data passed an internal review.

☰

Lineage

Sources

Area- and Depth-Weighted Averages of Selected SSURGO Variables for the Conterminous United States and District of Columbia

CC ID: 989549

**Contact Role Type:**	Originator
**Contact Type:**	Organization
**Contact Name:**	U.S. Department of Agriculture, Natural Resources Conservation Service
**Citation URL:**	https://water.usgs.gov/lookup/getspatial?ds866_ssurgo_variables
**Source Contribution:**	This digital data release consists of seven national data files of area- and depth-weighted averages of select soil attributes for every available county in the conterminous United States and the District of Columbia as of March 2014. The files are derived from Natural Resources Conservations Service'€™s (NRCS) Soil Survey Geographic database (SSURGO). The data files can be linked to the raster datasets of soil mapping unit identifiers (MUKEY) available through the NRCS'€™s Gridded Soil Survey Geographic (gSSURGO) database (http://www.n-rcs.usda.gov/wps/portal/nrcs/detail/soils/survey/geo/?cid=nrcs142p2_053628).

NOAA's Coastal Change Analysis Program (C-CAP) 2010 Regional Land Cover Data - Coastal United States

CC ID: 989551

**Contact Role Type:**	Originator
**Contact Type:**	Organization
**Contact Name:**	NOAA Office for Coastal Management
**Citation URL:**	https://coast.noaa.gov/digitalcoast/data/

**Source Contribution:**	The NOAA Coastal Change Analysis Program (C-CAP) produces national standardized land cover and change products for the coastal regions of the U.S. C-CAP products inventory coastal intertidal areas, wetlands, and adjacent uplands with the goal of monitoring changes in these habitats, on a one-to-five year repeat cycle. The timeframe for this metadata is reported as 2010-Era, but the actual dates of the Landsat imagery used to create the land cover may have been acquired a few years before or after each era. These maps are developed utilizing Landsat Thematic Mapper imagery, and can be used to track changes in the landscape through time. This trend information gives important feedback to managers on the success or failure of management policies and programs and aid in developing a scientific understanding of the Earth system and its response to natural and human-induced changes. This understanding allows for the prediction of impacts due to these changes and the assessment of their cumulative effects, helping coastal resource managers make more informed regional decisions. NOAA C-CAP is a contributing member to the Multi-Resolution Land Characteristics consortium and C-CAP products are included as the coastal expression of land cover within the National Land Cover Database.

USGS National Watershed Boundary Dataset

CC ID: 989550

**Contact Role Type:**	Originator
**Contact Type:**	Organization
**Contact Name:**	U.S. Geological Survey
**Citation URL:**	https://www.sciencebase.gov/
**Citation URL Description:**	https://water.usgs.gov/GIS/dsdl/Layer.zip
**Source Contribution:**	The Watershed Boundary Dataset (WBD) is a seamless, national hydrologic unit dataset. Simply put, hydrologic units represent the area of the landscape that drains to a portion of the stream network. More specifically, a hydrologic unit defines the areal extent of surface water drainage to an outlet point on a dendritic stream network or to multiple outlet points where the stream network is not dendritic. A hydrologic unit may represent all or only part of the total drainage area to an outlet point so that multiple hydrologic units may be required to define the entire drainage area at a given outlet. Hydrologic unit boundaries in the WBD are determined based on topographic, hydrologic, and other relevant landscape characteristics without regard for administrative, political, or jurisdictional boundaries. The WBD seamlessly represents hydrologic units at six required and two optional hierarchical levels.

Process Steps

Process Step 1

CC ID: 989537

Description:

Marsh Unit Codes (MUC) were created from the C-CAP 30 meter land cover data. Classes 16-18 (the estuarine wetland classes) were extracted and recoded using a morphometric algorithm to distinguish between different components of the marsh units. The following classification scheme was established:

gridcode1 = core wetland

gridcode2 = vegetated edge of wetland

gridcode3 = unvegetated edge of wetland

Core-to-edge ratio computed from MUCs and aggregated by 12-digit HUC. [Core_Edge_ratio = gridcode1 / (gridcode2 + gridcode3)]

Unvegetated edge to vegetated edge ratio was computed from MUCs and aggregated by 12-digit HUC. [UnvegVegEdge_ratio = gridcode3 / gridcode2]

A simplified version of the C-CAP 30 meter land cover was generated to facilitate derivation of land cover metrics. The resulting data layer had the following classification scheme:

gridcode2 = high intensity developed

gridcode3 = medium intensity developed

gridcode4 = low intensity developed

gridcode5 = open space developed

gridcode6 = agricultural classes (pasture/hay, cultivated)

gridcode7 = natural cover types (grassland, shrub, forest)

Percent impervious cover was computed from the simplified version of C-CAP 30 meter land cover data and aggregated by 12-digit HUCs. Analysis was performed within a 150 meter buffer around each marsh unit. [Perc_IC = ((gridcode2 * 0.8503) + (gridcode3 * 0.5768) + (gridcode4 * 0.2929) + (gridcode5 * 0.0941)) / Total_Area * 100]

Percent natural cover was computed from the simplified version of C-CAP 30 meter land cover data and aggregated by 12-digit HUCs. Analysis was performed within a 150 meter buffer around each marsh unit. [Perc_Natural = gridcode7 / Total_Area * 100]

Percent agricultural cover was computed from the simplified version of C-CAP 30 meter land cover data and aggregated by 12-digit HUCs. Analysis was per-

formed within a 150 meter buffer around each marsh unit. [Perc_Ag = gridcode6 / Total_Area * 100]

Soil erodibility was computed using Esri's USA Soils Erodibility Factor image service (https://landscape11.arcgis.com/arcgis/rest/services/USA_Soils_Erodibility_Factor/ImageServer, accessed March 2018). Analysis was performed within each marsh unit, not the entire HUC. The average erodibility factor for each marsh unit was weighted by the size of the marsh unit and aggregated by 12-digit HUC.

Tidal range was computed as the height difference between Mean Higher High Water (MHHW) and Mean Lower Low Water (MLLW) measured in meters. The tidal datum data were extracted from the VDatum tool and interpolated across data gaps to provide complete coverage within the study area.

Percent of marsh below mean higher high water (MHHW) was computed by intersecting all marsh units below MHHW, dividing by the total marsh area, and aggregating by 12-digit HUC.

Percent of marsh below mean tide level (MTL) was computed by intersecting all marsh units below MTL, dividing by the total marsh area, and aggregating by 12-digit HUC.

Percent hardened shoreline was computed using the Environmental Sensitivity Index (ESI) database. The ESI shoreline data were divided by and associated with 12-digit HUC codes. Within each HUC, all shoreline features that were armored (GENERALIZED_ESI_TYPE LIKE '%Armored%') were divided by the total shoreline length and multiplied by 100.

Environmental Sensitivity Index (ESI) maps provide a concise summary of coastal resources that are at risk if an oil spill occurs nearby. Examples of at-risk resources include biological resources (such as birds and shellfish beds), sensitive shorelines (such as marshes and tidal flats), and human-use resources (such as public beaches and parks).

Process Date/Time: 2020-09-01 00:00:00

Process Step 2

CC ID: 989548

Description: NOAA ESI National Shoreline (2017)

Migration space was determined using NOAA's sea level rise inundation data. Within every 12-digit HUC and for each foot of inundation above MHHW, the area of potential future marsh was divided by the current area of marsh to generate a ratio of future to present "potential" marsh area. This operation was performed for 1-6 feet of sea level rise inundation scenarios. For each SLR scenario, the resulting migration ratio values were ranked and scored by a quantile distribution function. The six scenarios were were then averaged, a

new quantile rank and score was generated and reported by AVG_migration_ratio.

Wetland connectedness, at this national level analysis, was computed using the marsh unit data (MUCs) and an analysis of projected fragmentation/consolidation under the 4 foot future sea level rise (SLR) scenario. A region grouping process was used to group all connected marsh units under current and future scenarios. Within each 12-digit HUC, the number of unique future marsh units were subtracted from the number of unique current marsh units, and divided by the number of unique current marsh units. This became the unitless raw value for the Wetland_Connectedness metric.

Shoreline sinuosity was assessed using the NOAA ESI shoreline vector data to characterize sinuosity within each 12-digit HUC. A sinuosity index was computed using a Sinuosity python script provided by Esri. High values represent linear shorelines and low values represent sinuous shorelines. Since the the sinuosity script generates values from 0-1, where 1 is a straight feature, we multiplied the sinuosity index by -1 so that values closer to 0 were scored higher in the quantile index.

Quantile scores were generated using a python script developed by NOAA OCM. Each metric was ranked and scored 1-10 based on the quantile position in the ranking. The quantile scores were then inversed if required based on the intent of the metric (negative or positive contributor to resilience). The quantile scores were summed by category (current condition, vulnerability, and adaptive capacity) and all combined, and new quantile rankings and scores were generated.

The numerical quantile scores were used to generate ordinal data describing the degree to which marsh units scored "low" or "high" for each resilience category (current condition, vulnerability, and adaptive capacity). Scores 1-5 were assigned "low" and scores 6-10 were assigned "high." An overall management category was generated using a concatenation of the three resilience categories, in the order just shown.

Vulnerability scores were updated to fix an error caused by the erroneous inclusion of the hardened shoreline metric in the original scoring (3/23/2022).

**Process Date/Time:**	2020-09-01 00:00:00

Catalog Details

**Catalog Item ID:**	62985
**GUID:**	gov.noaa.nmfs.inport:62985

**Metadata Record Created By:**	Erik Hund
**Metadata Record Created:**	2020-09-23 18:03+0000
**Metadata Record Last Modified By:**	SysAdmin InPortAdmin
**Metadata Record Last Modified:**	2022-08-09 17:11+0000
**Metadata Record Published:**	2020-10-02
**Owner Org:**	OCM
**Metadata Publication Status:**	Published Externally
**Do Not Publish?:**	N
**Metadata Last Review Date:**	2020-10-02
**Metadata Review Frequency:**	1 Year
**Metadata Next Review Date:**	2021-10-02
