# Supplementary material for: Marsh migration and beyond: A scalable framework to assess tidal wetland resilience and support strategic management
Source: PLoS One. 2023 Nov 6;18(11):e0293177. doi: 10.1371/journal.pone.0293177 (PMC10627444; doi:10.1371/journal.pone.0293177)
Supplement: S2 File — (PDF) [file pone.0293177.s002.pdf]

S2. Summary results for each spatial scale of application to US marshes
Table A. Abbreviations used for metrics, states, and National Estuarine Research Reserves

Full Name	Abbreviation
Metrics	
Area to edge ratio	Core_Edge_ratio
Unvegetated edge ratio	UnvegVegEdge_ratio
% impervious	Perc_IC
% natural	Perc_Natural
% agricultural	Perc_Ag
Soil erodibility	Erodibility_Factor
Tidal range	MEAN_Marsh_Tidal_Range
% marsh below MHHW	Pct_MUC_below_MHHW
% marsh below MTL	Mct_MUC_below_MTL
Hardened shoreline	Perc_Hardened_Shoreline
Shoreline complexity	Shoreline_Sinuosity
Marsh migration space	AVG_migration_ratio
Wetland "connectedness"	Wetland_Connectedness
States	
Alabama	AL
California	CA
Connecticut	CT
Deleware	DE
Florida	FL
Georgia	GA
Louisianna	LA
Maine	ME

Maryland	MD
Massachusetts	MA
Mississippi	MS
New Hampshire	NH
New Jersey	NJ
New York	NY
North Carolina	NC
Oregon	OR
Pennsylvania	PA
Rhode Island	RI
South Carolina	SC
Texas	TX
Virginia	VA
Washington	WA
National Estuarine Research Reserves (NERRs)	
Apalachicola National Estuarine Research Reserve	APA, FL
Ashepoo Combahee Edisto Basin National Estuarine Research Reserve	ACE, SC
Chesapeake Bay National Estuarine Research Reserve (Maryland)	CBM, MD
Chesapeake Bay National Estuarine Research Reserve (Virginia)	CBV, VA
Connecticut National Estuarine Research Reserve	CCT, CT
Delaware National Estuarine Research Reserve	DEL, DE
Elkhorn Slough National Estuarine Research Reserve	ELK, CA
Grand Bay National Estuarine Research Reserve	GND, MS
Great Bay National Estuarine Research Reserve	GRB, NH
Guana Tolomato Matanzas National Estuarine Research Reserve	GTM, FL
Hudson River National Estuarine Research Reserve	HUD, NY

Jacques Cousteau National Estuarine Research Reserve	JAC, NJ
Mission-Aransas National Estuarine Research Reserve	MAR, TX
Narragansett Bay National Estuarine Research Reserve	NAR, RI
North Carolina National Estuarine Research Reserve	NOC, NC
North Inlet-Winyah Bay National Estuarine Research Reserve	NIW, SC
Padilla Bay National Estuarine Research Reserve	PDB, WA
Rookery Bay National Estuarine Research Reserve	RKB, FL
San Francisco Bay National Estuarine Research Reserve	SFB, CA
Sapelo Island National Estuarine Research Reserve	SAP, GA
South Slough National Estuarine Research Reserve	SOS, OR
Tijuana River National Estuarine Research Reserve	TJR, CA
Waquoit Bay National Estuarine Research Reserve	WQB, MA
Weeks Bay National Estuarine Research Reserve	WKB, AL
Wells National Estuarine Research Reserve	WEL, ME

Table B. Tidal marsh resilience scores for the coastal contiguous US. Average resilience score (with standard deviation) by metric, metric category, and total marsh units analyzed within the scope of this study. Readers should note that the raw metric data were relativized to create metric resilience scores, so the nation-wide averages presented below are the averages of the distribution of scores between 1 and 10 for each metric.

	Average Resilience Score
Metrics	
Core Edge ratio	5.47 (2.91)
Perc IC	-5.5 (2.87)
Perc Natural	5.5 (2.87)
Perc Ag	-5.3 (3.12)
Erodibility Factor	-5.5 (2.87)
MEAN Marsh Tidal Range	5.5 (2.87)
Perc Hardened Shoreline	-5.42 (2.98)
Shoreline Sinuosity	5.5 (2.87)
Wetland Connectedness	5.45 (2.96)
Pct MUC below MHHW	-5.5 (2.87)
Pct MUC below MTL	-5.5 (2.88)
UnvegVegEdge ratio	-5.5 (2.87)
AVG migration ratio	5.5 (2.87)
Metric Categories	
Current Condition	5.65 (2.92)
Vulnerability	-5.32 (2.97)
Adaptive Capacity	5.65 (2.93)
Total	
Total Resilience	5.57 (2.91)

Table C. Tidal marsh resilience scores for regions within the coastal contiguous US. Average resilience score (with standard deviation) by metric, metric category, and total marsh units analyzed within the scope of this study.

	Gulf of Mexico	Mid-Atlantic	Northeast	Southeast	West Coast
Metrics					
Core Edge ratio	6.73 (2.82)	5.09 (2.76)	4.42 (2.32)	6.32 (2.87)	3.86 (2.52)
Perc IC	-4.96 (2.88)	-5.61 (2.81)	-6.69 (2.68)	-4.73 (2.63)	-6.21 (2.9)
Perc Natural	6.51 (2.82)	4.5 (2.47)	4.72 (2.75)	6.67 (2.44)	4.83 (3.12)
Perc Ag	-4.32 (2.92)	-6.86 (2.83)	-5.79 (2.59)	-4.72 (2.7)	-4.54 (3.54)
Erodibility Factor	-4.23 (2.96)	-5.72 (2.53)	-7.92 (2.22)	-3.88 (2.2)	-7.27 (1.97)
MEAN Marsh Tidal Range	3.17 (1.58)	5.43 (1.94)	8.77 (1.62)	4.55 (3.14)	8.24 (1.7)
Perc Hardened Shoreline	-5.12 (3.14)	-6.25 (2.92)	-5.57 (2.42)	-4.92 (2.45)	-4.92 (3.36)
Shoreline Sinuosity	6.41 (2.88)	5.33 (2.67)	4.62 (2.19)	5.99 (2.56)	4.38 (3.31)
Wetland Connectedness	5.66 (3.02)	5.63 (2.71)	4.58 (2.24)	7.04 (2.67)	3.74 (2.97)
Pct MUC below MHHW	-4.95 (2.55)	-6.04 (2.5)	-7.14 (2.11)	-5.88 (3.12)	-3.97 (3.21)
Pct MUC below MTL	-5.53 (2.9)	-6.08 (2.57)	-6.95 (2.58)	-5.19 (2.47)	-3.8 (3.06)
UnvegVegEdge ratio	-5.59 (3.1)	-5.91 (2.47)	-5.61 (2.83)	-4.7 (2.65)	-5.42 (3.2)
AVG migration ratio	5.18 (3.13)	5.38 (2.54)	5.11 (2.39)	6 (2.91)	5.98 (3.1)
Metric Categories					
Current Condition	6.9 (2.67)	4.44 (2.5)	4.46 (2.68)	7.08 (2.45)	4.94 (3.08)
Vulnerability	-5.58 (3.06)	-6 (2.71)	-6.48 (2.59)	-4.88 (2.59)	-3.45 (2.95)
Adaptive Capacity	5.81 (2.97)	5.16 (2.95)	4.83 (2.65)	6.98 (2.43)	5.36 (3.03)
Total					
Total Resilience	6.18 (2.87)	4.49 (2.66)	4.22 (2.62)	7.04 (2.65)	5.76 (2.82)

Table D. Tidal marsh resilience scores for states within the coastal contiguous US. Average resilience score (with standard deviation) by metric, metric category, and total marsh units analyzed within the scope of this study.

	AL	CA	CT	DE	FL	GA	LA	MA	MD	ME	MS	NC	NH	NJ	NY	OR	PA	RI	SC	TX	VA	WA
Metrics																						
Core Edge ratio	5.76 (2.33)	3.52 (2.53)	3.92 (2.55)	6.41 (2.66)	6.35 (2.66)	8.82 (2.19)	7.39 (2.94)	4.84 (2.2)	4.86 (2.48)	4.53 (2.3)	7.93 (1.73)	5.22 (2.83)	4.69 (2.93)	5.97 (3.26)	4.64 (2.36)	4.52 (2.56)	1.33 (0.5)	3.63 (1.46)	7.98 (1.74)	5.91 (2.89)	4.83 (2.66)	4.15 (2.42)
Perc IC	-5.36 (2.53)	-7.15 (2.91)	-8.62 (2.12)	-5.78 (2.3)	-6.16 (2.89)	-4.69 (2.08)	-4.22 (2.77)	-8.33 (1.74)	-4.42 (2.21)	-4.89 (2.14)	-5.55 (3.04)	-3.78 (2.29)	-6.08 (2.1)	-7.09 (2.51)	-8.6 (1.76)	-5.18 (2.56)	-6.44 (3.47)	-8.42 (2.09)	-4.54 (2.42)	-4.32 (2.39)	-4.21 (2.4)	-5.09 (2.44)
Perc Natural	6.52 (2.42)	3.4 (2.8)	2.62 (2.07)	3.55 (1.89)	5.74 (2.98)	7.49 (1.76)	7.17 (2.76)	3.22 (1.82)	4.61 (2.22)	6.65 (2.21)	6.21 (2.78)	7.2 (2.15)	4.15 (2.61)	4.12 (2.38)	2.76 (2.12)	6.84 (2.35)	4.56 (3.09)	3.1 (1.76)	6.85 (2.11)	6.28 (2.47)	5.84 (2.33)	6.36 (2.7)
Perc Ag	-4.68 (2.08)	-4.73 (3.72)	-4.88 (2.28)	-8.04 (2.68)	-3.04 (2.15)	-3.67 (1.53)	-4.62 (3.15)	-5.61 (2.28)	-8.02 (2.54)	-5.89 (2.79)	-4.72 (1.19)	-5.44 (2.9)	-7.23 (3)	-5.63 (2.78)	-4.96 (2.46)	-3.27 (3.11)	-5.11 (3.37)	-6.63 (1.95)	-5.47 (2.47)	-6.11 (3.12)	-7.16 (2.49)	-4.75 (3.32)
Erodibility Factor	-6.2 (1.83)	-6.36 (1.7)	-9.62 (0.628)	-5.02 (2.45)	-1.61 (1.1)	-3.44 (0.821)	-5.09 (2.68)	-5.69 (2.2)	-6.7 (2.2)	-8.64 (1.57)	-7.52 (2.75)	-4.61 (2.45)	-7.92 (1.98)	-3.31 (1.86)	-5.05 (3.01)	-8.16 (1.6)	-6.67 (3.04)	-6.21 (2.07)	-4.32 (1.11)	-6.79 (1.66)	-6.76 (1.56)	-8.39 (1.78)
MEAN Marsh Tidal Range	3.04 (0.539)	7.46 (1.37)	7.45 (1.26)	7 (1.43)	4.19 (1.77)	8.92 (0.354)	2.71 (1.15)	8.47 (1.77)	4.22 (0.868)	10 (0)	3.48 (0.509)	2.42 (1.95)	7.85 (1.34)	6.94 (1.91)	6.49 (1.84)	8.96 (0.211)	8.67 (0.5)	6.42 (0.507)	8.11 (0.767)	1.64 (0.68)	4.48 (1.47)	9.22 (1.89)
Perc Hardened Shoreline	-7.2 (2)	-4.22 (3.66)	-6.88 (2.6)	-5.06 (2.86)	-5.52 (3.34)	-3.74 (1.25)	-3.72 (2.53)	-6.57 (2.05)	-6.16 (3.03)	-4.18 (1.89)	-5.59 (2.72)	-5.27 (2.38)	-5.46 (1.33)	-5.75 (3.07)	-8 (2.39)	-5.46 (2.54)	-8.56 (1.81)	-7.68 (1.67)	-4.14 (1.42)	-6.27 (3.11)	-5.97 (2.62)	-5.84 (2.88)
Shoreline Sinuosity	5.28 (2.39)	4.42 (3.74)	5.08 (2.68)	6.71 (2.4)	5.85 (2.84)	7.51 (2)	7.65 (2.55)	4.55 (2.19)	5.09 (2.49)	4.49 (1.86)	6.21 (2.37)	5.36 (2.46)	5 (2.89)	5.93 (2.94)	3.48 (2.28)	4.54 (2.5)	3.78 (1.86)	4.32 (2.26)	7.27 (1.43)	5.26 (3.15)	5.84 (2.42)	4.24 (2.83)
Wetland Connectedness	7.48 (2.26)	3.03 (2.68)	4.15 (2.52)	7.55 (2.24)	7.11 (2.56)	7.33 (2.07)	3.62 (2.48)	4.57 (1.81)	5.98 (2.58)	4.69 (2.36)	6.1 (2.62)	6.99 (2.95)	5.15 (1.72)	6.02 (2.59)	3.2 (1.75)	4.48 (2.94)	3 (2.5)	4.53 (2.32)	8.15 (1.85)	5.13 (2.6)	5.86 (2.6)	4.6 (3.16)
Pct MUC below MHHW	-4.12 (1.76)	-2.11 (1.77)	-7 (2.72)	-6.94 (2.59)	-4.8 (2.32)	-7.8 (2.58)	-6.15 (2.59)	-7.61 (1.66)	-5.96 (2.21)	-7.2 (1.85)	-5.79 (2.11)	-4.67 (2.76)	-5.69 (2.56)	-6.7 (2.86)	-6.51 (2.13)	-5.43 (2.96)	-6.33 (2.78)	-6.84 (2.36)	-9.11 (1.23)	-2.87 (2.06)	-5.17 (2.44)	-6.42 (3.21)
Pct MUC below MTL	-4.64 (2.33)	-2.49 (2.06)	-6.9 (3.11)	-4.33 (2.58)	-4.74 (2.74)	-3.82 (1.39)	-6.9 (2.73)	-6.96 (2.37)	-6.84 (2.13)	-7 (2.44)	-6.52 (2.01)	-6.27 (2.48)	-5.08 (2.66)	-4.63 (2.42)	-7.96 (1.95)	-5.61 (2.66)	-6.56 (3.43)	-8.05 (2.12)	-4.03 (1.49)	-4.29 (2.68)	-5.71 (2.37)	-5.22 (3.55)
UnvegVegEdge ratio	-4.96 (2.01)	-5.56 (3.52)	-6.68 (2.96)	-4.31 (2.07)	-4.56 (2.68)	-4.74 (2.44)	-7.23 (3.2)	-6 (2.6)	-5.46 (2.34)	-5.13 (2.78)	-5.9 (2.74)	-4.19 (2.67)	-3.69 (2.53)	-6.4 (2.3)	-7.41 (2.24)	-5.91 (2.31)	-7.11 (2.76)	-6.21 (2.64)	-5.74 (2.03)	-4.93 (2.89)	-5.71 (2.45)	-5 (2.94)
AVG migration ratio	6.68 (1.93)	4.82 (3.03)	4.5 (2.51)	5.14 (2.22)	6.06 (2.48)	4.64 (3.22)	3.67 (3.5)	3.94 (1.96)	5.81 (2.14)	6.04 (2.26)	4.34 (2.37)	7.1 (2.71)	5.31 (2.43)	5.16 (2.81)	3.3 (1.77)	7.02 (2.77)	8.11 (1.05)	4.32 (1.97)	4.74 (2.4)	5.57 (3.02)	6.09 (2.58)	7.45 (2.51)
Metric Categories																						
Current Condition	6.48 (2.66)	3.85 (2.8)	2.78 (2.26)	4.69 (1.88)	6.75 (2.8)	8.56 (1.52)	7.04 (2.67)	3.33 (2.04)	4.59 (2.46)	6.04 (2.32)	6.93 (2.79)	7.07 (2.51)	4.77 (2.68)	4.26 (2.4)	2.57 (1.95)	6.57 (2.73)	2.89 (2.52)	2.37 (1.8)	7.36 (1.85)	6.5 (2.39)	5.43 (2.47)	6.06 (2.99)
Vulnerability	-5.8 (2.53)	-1.89 (1.48)	-7.78 (2.44)	-4.29 (2.16)	-3.35 (2.28)	-2.77 (1.35)	-7.47 (2.49)	-5.69 (2.36)	-7.49 (2.12)	-6.35 (2.59)	-8.07 (2.63)	-6.19 (2.62)	-5.31 (2.39)	-3.65 (2.18)	-6.34 (2.56)	-5.14 (2.89)	-4.89 (3.33)	-7.32 (2.5)	-4.27 (1.42)	-5.86 (2.37)	-6.36 (2.5)	-5.34 (3.29)
Adaptive Capacity	6.4 (2.04)	4.73 (3.31)	3.82 (2.74)	6.59 (2.64)	6.47 (2.99)	7.26 (1.96)	5.12 (2.91)	3.35 (2.13)	5.45 (2.78)	6.24 (2.24)	5.24 (2.46)	7.35 (2.15)	5.23 (1.69)	5.36 (3.1)	1.93 (1.79)	6.07 (2.59)	4.44 (1.67)	3.05 (2.35)	7.41 (1.98)	5.12 (3.02)	6.04 (2.46)	6.11 (2.44)
Total																						
Total Resilience	5.96 (2.59)	5.41 (2.92)	2.78 (2.2)	6.04 (2.12)	7.09 (2.85)	8.54 (1.45)	5.44 (2.8)	3.24 (2.13)	4.19 (2.53)	5.58 (2.36)	5.14 (2.88)	6.8 (2.75)	4.69 (2.32)	5.39 (2.58)	1.95 (1.7)	6.39 (2.37)	3.44 (2.07)	2.21 (2.25)	7.64 (1.92)	5.45 (2.54)	5.15 (2.46)	6.06 (2.76)

Table E. Tidal marsh resilience scores for National Estuarine Research Reserves (NERRs) within the coastal contiguous US. Average resilience score (with standard deviation) is provided by metric, metric category, and total marsh units analyzed within the scope of this study. Scores for NERRs which only contain a single MUC are listed without standard deviations.

	ACE, SC	APA, FL	CBM, MD	CBV, VA	CCT, CT	DEL, DE	ELK, CA	GND, MS	GRB, NH	GTM, FL	HUD, NY	JAC, NJ	MAR, TX	NAR, RI	NIW, SC	NOC, NC	PDB, WA
Metrics																	
Core Edge ratio	9 (1.35)	6 (2.83)	7.75 (1.5)	6.6 (2.41)	5.17 (2.14)	9.5 (0.707)	7	10 (0)	3.6 (1.67)	6.33 (1.37)	6 (0)	8.75 (2.6)	7.53 (1.12)	3.67 (0.577)	9.5 (0.707)	7 (1.23)	3.5 (0.707)
Perc IC	-3.17 (1.47)	-3.83 (2.93)	-4.25 (1.5)	-5.2 (2.17)	-8.67 (1.03)	-4 (2.83)	-8	-6 (4.24)	-5.8 (0.447)	-6.67 (1.97)	-8.5 (0.707)	-6.92 (2.78)	-4.07 (1.62)	-8.33 (0.577)	-3.5 (3.54)	-7.2 (1.64)	-7.5 (2.12)
Perc Natural	8.33 (1.23)	8.5 (2.07)	5.75 (1.5)	5.6 (1.82)	3.17 (1.17)	2 (0)	2	6.5 (3.54)	3.4 (1.34)	6 (2.37)	4 (1.41)	5.58 (2.64)	6.67 (2.38)	3.67 (1.53)	8.5 (2.12)	4.6 (1.82)	2.5 (0.707)
Perc Ag	-5.42 (2.39)	-2.83 (1.47)	-8.25 (1.71)	-7.6 (0.894)	-6.17 (0.983)	-10 (0)	-10	-2.5 (2.12)	-9.2 (0.447)	-3.83 (0.753)	-6.5 (2.12)	-4.17 (2.04)	-6.13 (3.23)	-7.67 (0.577)	-1 (0)	-6.4 (1.82)	-9.5 (0.707)
Erodibility Factor	-4.67 (0.985)	-1.33 (0.516)	-6.75 (1.71)	-6.6 (0.548)	-9.17 (0.408)	-5.5 (0.707)	-7	-8.5 (0.707)	-7.6 (1.82)	-1 (0)	-8.5 (0.707)	-1.83 (0.577)	-6.53 (1.36)	-3.67 (0.577)	-2.5 (0.707)	-2.6 (1.52)	-9.5 (0.707)
MEAN Marsh Tidal Range	8.17 (0.389)	3.67 (1.37)	4.5 (0.577)	5.6 (0.548)	6.17 (0.408)	8 (0)	7	3 (0)	7 (0)	6.17 (1.17)	6 (0)	5.5 (0.905)	1.07 (0.258)	7 (0)	7 (0)	5.6 (2.61)	9 (0)
Perc Hardened Shoreline	-2.92 (1.24)	-3.33 (2.73)	-4.5 (3.11)	-6.6 (2.07)	-8.33 (1.21)	-2.5 (0.707)	-7	-5 (2.83)	-4.8 (0.837)	-5 (3.35)	-9.5 (0.707)	-5.67 (2.71)	-6.73 (1.62)	-8.33 (0.577)	-5.5 (0.707)	-6.8 (1.3)	-9.5 (0.707)
Shoreline Sinuosity	7.83 (1.27)	4.5 (1.64)	5 (4.08)	6.6 (2.07)	5.17 (1.6)	8.5 (0.707)	7	8.5 (0.707)	3.4 (1.95)	6 (2.45)	2.5 (0.707)	6.17 (2.59)	5.87 (2.36)	2.67 (1.53)	7 (1.41)	8 (0.707)	4.5 (2.12)
Wetland Connectedness	9 (1.13)	9 (0.894)	7.75 (3.3)	6.8 (0.447)	4.5 (1.52)	8 (1.41)	6	6.5 (2.12)	5 (1.23)	5.5 (2.43)	3 (0)	5.67 (2.5)	5.6 (1.8)	5 (0)	9.5 (0.707)	5.8 (1.3)	3.5 (0.707)
Pct MUC below MHHW	-9.17 (1.12)	-3.33 (1.51)	-5 (2.31)	-4.2 (1.1)	-6.5 (1.38)	-7 (0)	-2	-3 (0)	-5.2 (2.28)	-6.5 (3.27)	-6 (0)	-6.33 (3.08)	-2 (0.756)	-6.67 (0.577)	-9.5 (0.707)	-8.4 (3.05)	-9 (1.41)
Pct MUC below MTL	-3.58 (0.9)	-3.33 (2.88)	-5.5 (3)	-4.4 (1.52)	-7.17 (1.72)	-2 (0)	-3	-4 (0)	-6.2 (2.39)	-3 (1.79)	-9 (0)	-3.5 (1.09)	-3.4 (1.6)	-7.67 (0.577)	-4.5 (2.12)	-7.8 (1.64)	-10 (0)
UnvegVegEdge ratio	-5 (1.81)	-3.83 (2.56)	-4.5 (1.29)	-5.4 (2.61)	-6.67 (2.07)	-3.5 (0.707)	-6	-7 (1.41)	-3.6 (3.65)	-4 (2.53)	-6.5 (0.707)	-5.08 (2.81)	-3.73 (2.55)	-6 (1)	-5 (2.83)	-8.2 (0.837)	-7.5 (2.12)
AVG migration ratio	4 (2.41)	8.17 (1.6)	4.25 (0.957)	4.6 (2.19)	3.67 (1.63)	2.5 (0.707)	5	4 (0)	5.6 (2.07)	4.67 (1.21)	1.5 (0.707)	3.5 (2.54)	4 (2.39)	4.33 (1.16)	3.5 (0.707)	3.2 (1.3)	9 (1.41)
Metric Categories																	
Current Condition	8.75 (1.06)	8.5 (2.26)	6.75 (0.5)	5.6 (2.61)	2.67 (1.63)	6 (1.41)	2	7.5 (3.54)	3.8 (1.79)	6.83 (2.04)	3.5 (2.12)	7 (2.66)	7.6 (2.06)	2 (1)	9.5 (0.707)	3.8 (1.92)	1.5 (0.707)
Vulnerability	-4.25 (1.14)	-2.33 (2.34)	-5.75 (3.86)	-4.4 (1.14)	-8.17 (0.983)	-2.5 (0.707)	-2	-6.5 (0.707)	-6 (2.55)	-2 (0.632)	-8.5 (0.707)	-2.67 (0.985)	-5.2 (1.47)	-5.33 (1.16)	-4.5 (2.12)	-6.8 (1.1)	-10 (0)
Adaptive Capacity	8 (1.6)	9 (1.1)	6 (3.56)	5.2 (1.1)	2.33 (0.816)	7.5 (0.707)	5	6 (2.83)	5.2 (1.64)	5.67 (2.94)	1 (0)	4 (3.44)	4.2 (2.68)	2 (1)	6.5 (0.707)	4 (1.73)	5 (1.41)
Total																	
Total Resilience	8.58 (1.24)	9.5 (0.837)	6 (3.16)	5.4 (1.95)	1.67 (0.816)	7.5 (0.707)	4	6.5 (3.54)	3.8 (1.64)	7 (2.61)	1 (0)	6.17 (2.72)	5.87 (2.3)	1.67 (1.16)	9 (1.41)	3.2 (1.64)	1.5 (0.707)
