# Supplementary material for: Marsh migration and beyond: A scalable framework to assess tidal wetland resilience and support strategic management
Source: PLoS One. 2023 Nov 6;18(11):e0293177. doi: 10.1371/journal.pone.0293177 (PMC10627444; doi:10.1371/journal.pone.0293177)
Supplement: S3 File — (PDF) [file pone.0293177.s003.pdf]

S3. Case Study: Parcel Scale Planning in New Hampshire

Downscaling a broadly applicable framework can be helpful to support parcel-level management planning, the scale at which most on the ground restoration and conservation projects are implemented. Marsh Units within our framework can be created at any scale, as demonstrated by a consortium that implemented this framework in New Hampshire. Partners including Great Bay National Estuarine Research Reserve, New Hampshire Coastal Program, University of New Hampshire, and The Nature Conservancy subdivided 13 of the marsh units used in the national analysis into 224 new marsh units using natural breaks in high resolution (< 1 m) tidal wetland land cover data. These land cover data were created by the National Oceanic and Atmospheric Administration's (NOAA's) Office for Coastal Management and can be downloaded via NOAA's Digital Coast (coast.noaa.gov/digitalcoast/data/ccapsalthabitat.html). The average area of the newly created local marsh units was 26.13 acres. While marsh units in our national scale application of the framework contained emergent marshes in addition to their associated watersheds, the marsh units in this case study contained emergent marsh only.

The users then calculated resilience scores for each of the 224 marsh units using localized data sets for the same 13 metrics outlined in the main text, along with 6 additional metrics for current condition including ditch density, berm presence, and presence of invasive *Phragmites australis*. The additional metrics were developed with partners to address the locally relevant drivers of resilience given available data. In addition to informing best management options for a specific Marsh Unit (Table 1 in main text), resilience scores can be used to spatially plan the best locations for specific management techniques. For example, a practitioner interested in implementing a thin layer placement project could screen their geography of interest to select areas with high unvegetated to vegetated ratio to further assess in the field.

In this real-world example, a local conservation group was interested where best to locate land protection projects. The users assessed resilience scores for marsh units within unprotected parcels of marsh and migration space in three towns of interest, and then opted to extend the framework to include a conservation score. Marsh units with high current condition, low vulnerability to RSLR, and high adaptation potential (Fig. 1, main text) were scored high for conservation (5). On the other end of the spectrum, those with low current condition, high vulnerability to RSLR, and low adaptation potential scored 0. Those with one marsh resilience category to mitigate receive a score of 3 and those with two categories to mitigate are scored 1. No marshes received a score of 2 or 4. This prioritization allowed the municipalities to focus where to invest landowner outreach and investment of conservation dollars strategically (Fig. S1). More information about this project can be found at https://greatbay.org/salt-marsh-plan/.

Figure S1. The number of local scale marsh units in each national scale marsh unit in New Hampshire and example spatial tax parcel prioritization for land protection based on the total resilience score. Imagery source: Esri, HERE, Gamin, (c) OpenStreetMap contributors, and the GIS user community.

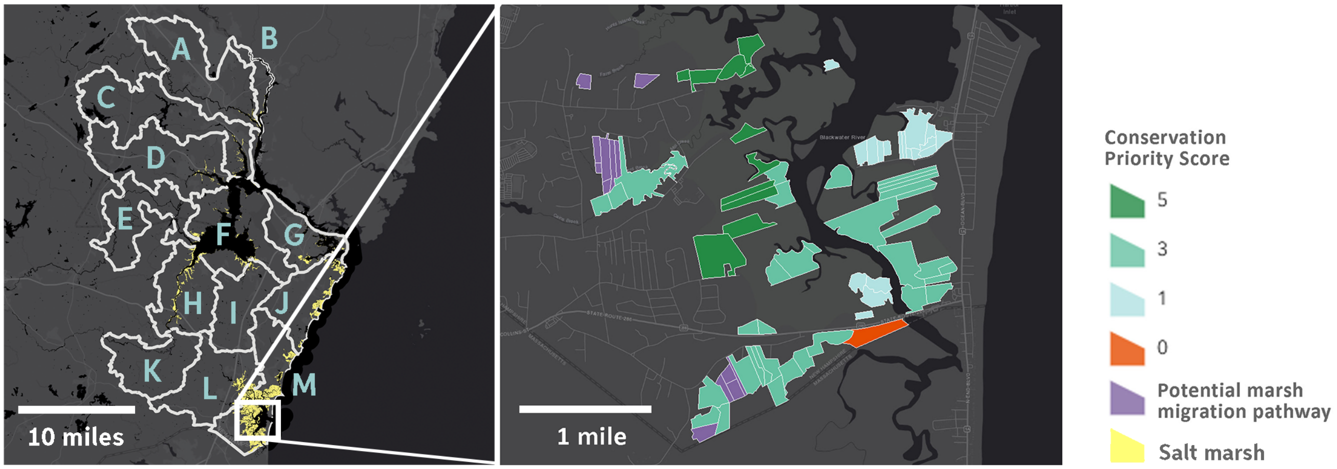

National Marsh Unit	A	B	C	D	E	F	G	H	I	J	K	L	M	Total
Number Local Marsh Units	7	4	9	17	3	37	49	19	3	20	0	12	44	224
